# Severe Splenic Injuries in Patients With Multiple Trauma

**DOI:** 10.1001/jamasurg.2026.0016

**Published:** 2026-02-25

**Authors:** Wei Huang, Caitlyn Braschi, Feifei Jin, Meghan Lewis, Demetrios Demetriades

**Affiliations:** 1 Peking University People’s Hospital, Trauma Center, Beijing, China; 2 Division of Trauma and Acute Care Surgery, Department of Surgery, Los Angeles General Medical Center, Los Angeles, California

## Abstract

**Importance:**

The optimal management of severe blunt splenic injuries (BSI) in patients with multiple trauma is debated. This study compares early outcomes of the 3 primary treatment approaches.

**Objective:**

To study the treatment patterns of severe BSI and characterize clinical outcomes in patients with multiple trauma.

**Design, Setting, and Participants:**

In this cohort study, adult patients with severe BSI were identified in the American College of Surgeons Trauma Quality Improvement Program database and excluded if the Abbreviated Injury Scale score was 2 or less for all body regions outside the abdomen. Outcomes were compared based on treatment approach. Subgroup analyses were performed in patients presenting with hypotension, normotension, and those whose initial nonoperative management (NOM) failed. The associations between intervention patterns and mortality, complications, and hospital course were examined. The database was queried for data from January 2017 to December 2022; data analysis was performed from September 2024 to January 2025.

**Exposures:**

Open splenectomy (OS), splenic angioembolization (SAE), or observation (OBS).

**Main Outcomes and Measures:**

The primary outcome was in-hospital mortality. Secondary outcomes included a variety of complications that included acute respiratory distress syndrome (ARDS), cardiac arrest, and severe sepsis, as well as hospital and intensive care unit length of stay (LOS).

**Results:**

In total, 12 930 patients with multiple trauma met the inclusion criteria (median [IQR] age, 39 [26-56] years; 9259 males [71.6%] and 3671 females [28.4%]). There were 3390 patients (26.2%) who underwent OS, 2537 (19.6%) who underwent SAE, and 7003 (54.2%) in the OBS group. Multivariable regression analysis found mortality risk, compared with the OS group, was lower for SAE (hazard ratio [HR], 0.62; 95% CI, 0.49 to 0.80; *P* < .001) and OBS (HR, 0.61; 95% CI, 0.50 to 0.74; *P* < .001). SAE and OBS had fewer complications compared with OS in the overall cohort (odds ratio [OR], 0.74; 95% CI, 0.64 to 0.86; *P* < .001, and OR, 0.75; 95% CI, 0.66 to 0.85; *P* < .001, respectively). For specific complications, the OS group had more ARDS, cardiac arrest, and severe sepsis. SAE and OBS had shorter hospital LOS (β, −1.37; 95% CI, −2.03 to −0.71; *P* < .001, and β, −1.33; 95% CI, −1.93 to −0.74; *P* < .001, respectively) and intensive care unit LOS (β, −1.42; 95% CI, −1.87 to −0.96; *P* < .001, and β, −1.34; 95% CI, −1.75 to −0.92; *P* < .001, respectively). The hypotensive subgroup had no increase in mortality, complications, or hospital course. Patients for whom NOM failed had more complications compared with upfront OS (OR, 3.09; 95% CI, 2.22 to 4.30; *P* < .001, and OR, 1.46; 95% CI, 1.21 to 1.76; *P* < .001, respectively). Sensitivity analysis confirmed these associations.

**Conclusions and Relevance:**

This study found that splenic salvage in patients with multiple trauma was associated with decreased mortality, fewer complications, and shorter hospital course compared with splenectomy. NOM in patients presenting with hypotension showed that outcomes were not inferior, while the failure of NOM was associated with more complications. NOM should be attempted, even in patients with multiple trauma who present with hypotension.

## Key Points

**Question:**

What is the optimal approach to manage severe blunt splenic injury in patients with multiple trauma?

**Findings:**

In this cohort study that included 12 930 patients, nonoperative management (angioembolization or observation) was associated with a reduction in mortality, morbidity, and hospital course compared with splenectomy in patients with multiple trauma. Among patients with hypotension on admission, nonoperative management showed no increase in mortality, morbidity, or hospital course, but patients for whom nonoperative management failed had more complications.

**Meaning:**

These findings underscore that splenic salvage with nonoperative management may be preferred in patients with multiple trauma and high-grade splenic injury, but the selection of patients for nonoperative management should be done carefully.

## Teaser

This cohort study examines data from the American College of Surgeons Trauma Quality Improvement Program to analyze the treatment patterns in patients with severe blunt splenic injuries and characterize clinical outcomes.

## Introduction

Blunt splenic injury (BSI) is common after abdominal trauma, occurring in up to 30% of patients with multiple trauma, with high-grade injuries posing significant management challenges.^1^ Historically, open splenectomy (OS) was the standard treatment for severe BSI, but nonoperative management (NOM), including observation (OBS) and splenic angioembolization (SAE), has gained traction because of its potential to reduce morbidity and preserve immunologic function.^2,3,4^ Despite this shift, the optimal approach for patients with multiple trauma, particularly those with hemodynamic instability or severe concomitant injuries, remains debated.^2,4,5,6^

Current guidelines advocate for NOM in hemodynamically stable patients, but evidence supporting its safety in patients with hypotension or multiple trauma is limited.^7^ Recent studies suggest SAE may improve outcomes in high-grade injuries, yet failure rates of more than 20% raise concerns about delayed complications.^8,9^ Furthermore, existing literature often excludes patients with multiple trauma, leaving gaps in understanding the interplay between splenic injury and systemic trauma burden.^3^

This study leverages the American College of Surgeons Trauma Quality Improvement Program (ACS-TQIP) database to compare outcomes of OS, SAE, and OBS in patients with multiple trauma and severe BSI. We hypothesize that SAE and OBS will reduce mortality and complications compared to OS, even in hypotensive subgroups. Our findings aim to refine evidence-based strategies for this high-risk population.

## Methods

### Data Source

This study was approved by the University of Southern California Ethics Committee and adheres to the Strengthening the Reporting of Observational Studies in Epidemiology (STROBE) reporting guidelines. The ACS-TQIP database was queried for data from January 2017 to December 2022. In 2022, the database contained information from 816 trauma centers.

### Participants

All adult patients with multiple trauma (aged ≥16 years) with severe BSI were included. Severe BSI was defined as an Abbreviated Injury Scale (AIS) score of 3, 4, or 5. Exclusion criteria included the following: AIS score of 2 or less for all body regions other than the abdomen, severe nonsplenic intra-abdominal solid organ injury (AIS score ≥3), abdominal hollow viscus injury, named abdominal vascular injury, transferred from another facility, died in the emergency department, had a hospital length of stay (HLOS) of 24 hours or less, left against medical advice, underwent laparoscopic surgery or splenic repair, or missing data for above exclusion criteria, age, sex, Injury Severity Score, systolic blood pressure (SBP), heart rate, respiratory rate, Glasgow Coma Scale score, comorbidities, complications, and splenic procedure time.

### Independent Variables

Demographic data, including age, sex, race, and body mass index, were obtained from the database. Hospital-related data included hospital type (nonprofit, profit, government), payer, bed size, and trauma center level. We captured emergency department vital signs (SBP, heart rate, respiratory rate, Glasgow Coma Scale score, temperature, pulse oximetry, respiratory assistance) and injury characteristics (Injury Severity Score, AIS score, mechanism). Comorbidities included alcohol use disorder, bleeding disorder, chemotherapy, congestive heart failure, smoking, chronic kidney failure, stroke, diabetes, hypertension, chronic obstructive pulmonary disease, steroid use, cirrhosis, dementia, anticoagulant therapy, angina pectoris, mental disorder, myocardial infarction, peripheral arterial disease, and substance abuse disorder.

### Outcomes

The primary outcome was in-hospital mortality. Secondary outcomes included any complications, specific complications (acute kidney injury, acute respiratory distress syndrome [ARDS], cardiac arrest, surgical site infection, severe sepsis, venous thromboembolism, deep vein thrombosis, pulmonary embolism, myocardial infarction, stroke, unplanned intubation, unplanned admission to the intensive care unit (ICU), unplanned visit to the operating room, catheter-associated urinary tract infection, central line–associated bloodstream infection, ventilator-associated pneumonia, alcohol withdrawal syndrome, and pressure ulcer, hospital course (HLOS, intensive care unit length of stay [ICULOS], ventilator time), and transfusion in 4 hours (packed red blood cell, plasma, platelet).

### Treatment Definitions

Patients were stratified by treatment patterns. We used 12 hours of admission as a time cutoff for grouping. Primary management was classified as open splenectomy (OS) (*International Statistical Classification of Diseases and Related Health Problems, Tenth Revision *[*ICD-10*], codes 07BP0ZZ and 07TP0ZZ), splenic angioembolization (SAE) (*ICD-10* codes 04L43DZ, 04L43ZZ, 04V43DZ, and 04V43ZZ), or an observation (OBS) group. OBS was defined as no OS or SAE within 12 hours of hospital admission. Failure was defined as the need for OS or SAE after primary management.

### Statistical Analysis

Continuous variables with normal distribution are presented as means (SD) and analyzed using a *t* test. Non–normally distributed continuous variables are reported as medians (IQR), and they were compared using the Mann-Whitney *U* test. Categorical variables are expressed as percentages, with their statistical significance determined by the χ^2^ test or Fisher exact test. We used Kaplan-Meier curves to estimate the cumulative incidence of in-hospital mortality, with differences assessed using the log-rank test.

Subgroup analysis was performed for patients presenting with hypotension (SBP <90 mm Hg) and normotension (SBP ≥90 mm Hg), those with a shock index 1 or more and shock index less than 1, and according to splenic injury severity. A second subgroup analysis compared outcomes of initial OS with those for whom SAE and OBS failed.

All statistical analyses and graphical representations were performed using RStudio version 4.4.1 (Posit PBC), with statistical significance defined as *P* < .05. Data analysis was performed from September 2024 to January 2025.

#### Main Analysis

To control for confounders, we performed multivariable Cox regression analyses to assess the risk of SAE and OBS compared with the OS group; hazard ratios (HRs) and 95% CIs were calculated. For other binary outcomes, multivariable logistic regression analyses were used to evaluate risks, with odds ratios (ORs) and 95% CIs calculated. For hospital course and transfusion, multivariable linear regression analyses were used to explore differences among groups, yielding regression coefficients (β) and 95% CI. Univariable and multivariable logistic regression analyses were used to identify risk factors of failure, with ORs and 95% CIs calculated. Potential confounders were entered into the model based on univariable analysis demonstrating *P* < .20. The adjusted variables are detailed in eTable 1 in Supplement 1.

#### Sensitivity Analysis

Inverse probability weighting was used as a sensitivity analysis to address potential selection bias where baseline characteristics were balanced among the OS, SAE, and OBS groups. We calculated propensity scores using logistic regression, incorporating all clinically relevant covariates, applied stabilized weights to minimize variance, and assessed intergroup balance using standardized mean differences (<0.1). Subsequently, the survey package was used to conduct weighted Cox regression, weighted logistic regression, and weighted linear regression analyses for the outcomes. Furthermore, we used 4 and 8 hours of admission as time cutoffs for grouping, and multivariable regression analysis was conducted as sensitivity analysis.

## Results

### Baseline Characteristics of Overall Cohort and Subgroups

**Figure 1.**
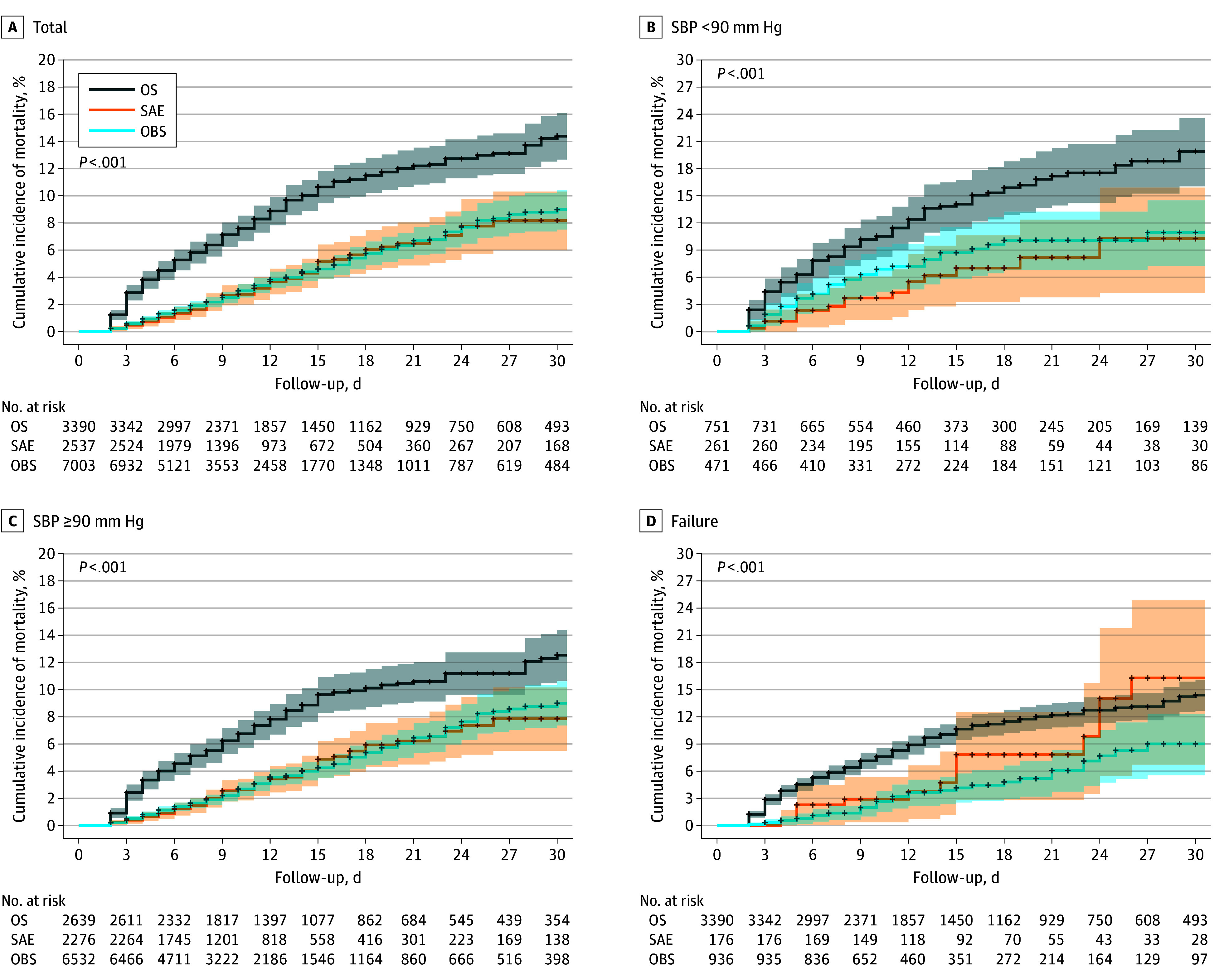
Cumulative Incidence of Mortality in the Open Splenectomy (OS), Splenic Angioembolization (SAE), and Observation (OBS) Groups Kaplan-Meier curves estimated cumulative incidence of 30-day mortality for the entire cohort (A) and 3 subgroups. B, The hypotension subgroup was patients with systolic blood pressure (SBP) less than 90 mm Hg and normotension subgroup (C), those with SBP of 90 mm Hg or greater. D, The failure subgroup compared outcomes of patients for whom SAE and OBS failed with initial OS.

In total, 12 930 patients with multiple trauma met our inclusion criteria (median [IQR] age, 39 [26-56] years; 9259 men [71.6%] and 3671 women [28.4%]). There were 3390 patients (26.2%) who underwent OS, 2537 (19.6%) who underwent SAE, and 7003 (54.2%) in the OBS group (eFigure in Supplement 1). The demographic, injury, comorbidity and hospital variables of the cohort and subgroups were shown in eTables 2, 4, 6, and 8 in Supplement 1. In our multitrauma cohort, the AIS score for the chest was 3; the chest was the most severely injured region outside of abdomen (eTable 2 in Supplement 1). The overall mortality rate was 686 of 12 930 patients (5.3%); it was highest in the OS group (344/3390; 10.1%) compared with SAE (88/2537; 3.5%) and OBS (254/7003; 3.6%; *P* < .001) (eTable 3 in Supplement 1). Estimated cumulative incidence of 30-day mortality is displayed in Kaplan-Meir curves (Figure 1), which demonstrate significantly higher mortality in the OS group in the cohort and in each subgroup. The overall complication rate was 2549 patients (19.7%) and highest in OS (989; 29.2%) compared with the SAE (441; 17.4%) and OBS groups (1119; 16.0%; *P* < .001) (eTable 3 in Supplement 1). The failure rate was 22 patients (0.6%) in the OS group, 176 patients (6.9%) in SAE, and 254 (13.4%) in OBS (*P* < .001) (eTable 3 in Supplement 1).

In the subgroup analysis of 1483 patients with hypotension, 751 (50.5%) underwent OS. The overall mortality rate was 174 of 1483 patients (11.7%) and highest in OS (114/751; 15.2%) compared with SAE (16/261; 6.1%) and OBS (44/471; 9.3%; *P* < .001) (eTable 5 in Supplement 1). The overall complication rate was 475 patients (32.0%) and highest in OS (268; 35.7%) compared with SAE (67; 25.7%), and OBS (140; 29.7%; *P* < .001). eTable 7 in Supplement 1 contains outcomes for the subgroup analysis of patients with normotension.

In the subgroup analysis of patients for whom their initial treatment failed, the mortality rate was 15 of 176 patients (8.5%) in the SAE group and 42 of 936 (4.5%) in OBS. The complication rate increased to 86 patients (48.9%) in SAE and 286 (30.6%) in OBS (eTable 9 in Supplement 1).

### Multivariable Cox Regression Analysis for Mortality

**Figure 2.**
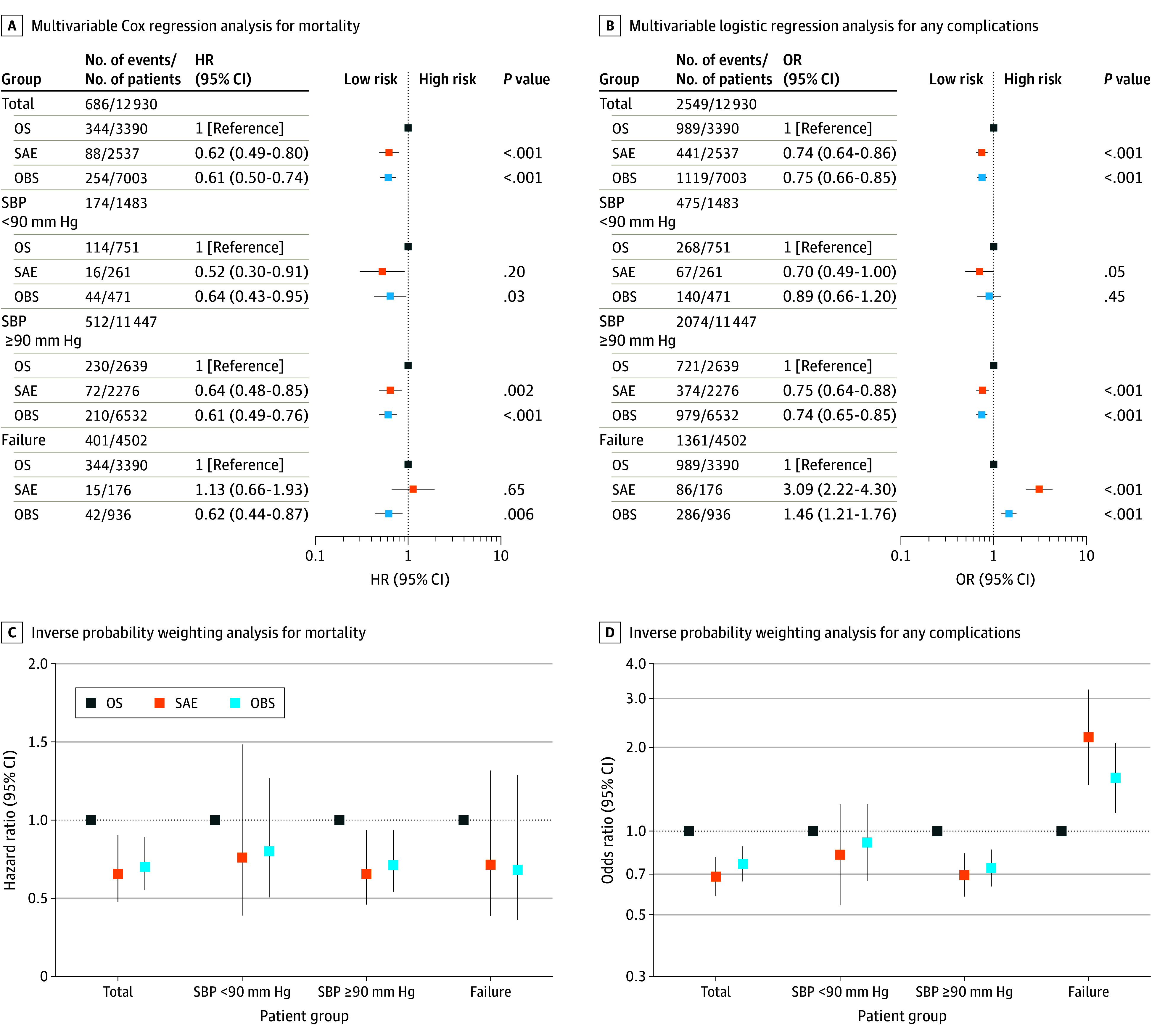
Multivariable Analysis for Mortality and Any Complications Plots reflect multivariable Cox regression analysis for mortality (A), multivariable logistic regression analysis for any complications (B), and inverse probability weighting analysis for mortality (C) and any complications (D). Hazard ratios (HRs) were determined for the risk of splenic angioembolization (SAE) and observation (OBS) compared with open splenectomy (OS) regarding mortality; odds ratios (ORs) were determined for the risk of SAE and OBS compared with OS regarding any complications. Failure refers to the subgroup comparing outcomes of patients for whom SAE and OBS failed with initial OS. SBP indicates systolic blood pressure.

Results of multivariable Cox regression analysis for mortality are shown in Figure 2A. Overall, compared with the OS group, the mortality risk for the SAE and OBS groups was significantly lower (HR, 0.62, 95% CI, 0.49-0.80; *P* < .001; and HR, 0.61; 95% CI, 0.50-0.74; *P* < .001, respectively). In the hypotension subgroup, there was also significantly lower mortality among SAE and OBS patients compared with OS (HR, 0.52; 95% CI, 0.30-0.91; *P* = .02; and HR, 0.64, 95% CI, 0.43-0.95; *P* = .03, respectively). In the normotension subgroup, the mortality risk was 0.64 (95% CI, 0.48-0.85; *P* = .002) in SAE and 0.61 (95% CI, 0.49-0.76; *P* < .001) in OBS, when compared with OS. In the failure subgroup, there was no significant difference in mortality risk in patients who underwent SAE (HR, 1.13; 95% CI, 0.66-1.93; *P* = .65), but OBS still had lower mortality risk (HR, 0.62; 95% CI, 0.44-0.87; *P* = .006).

There was also significantly lower mortality risk among SAE and OBS compared with OS in the subgroups shock index less than 1, shock index 1 or more, spleen AIS score 3, and spleen AIS score 4 (eTable 10 in Supplement 1).

### Multivariable Logistic Regression Analyses for Complications

Figure 2B summarizes the effect size for any complication. Overall, the risk of any complication was significantly lower after SAE (OR, 0.74; 95% CI, 0.64-0.86; *P* < .001) and OBS (OR, 0.75; 95% CI, 0.66-0.85; *P* < .001). This trend was also observed in the normotension subgroup. In the hypotension subgroup, the risk of any complication was significantly lower in SAE (OR, 0.70; 95% CI, 0.49-1.00; *P* = .047), but there was no difference in OBS. However, in the failure subgroup, the risk of any complication increased significantly in SAE (OR, 3.09; 95% CI, 2.22-4.30; *P* < .001) and in OBS (OR, 1.46; 95% CI, 1.21-1.76; *P* < .001).

Table 1 showed the effect size regarding specific complications. The risk of ARDS (OR, 0.47; 95% CI, 0.30-0.75; *P* = .001 for SAE; OR, 0.61; 95% CI, 0.43-0.86; *P* = .006 for OBS), cardiac arrest (OR, 0.53; 95% CI, 0.35-0.81; *P* = .003 for SAE; OR, 0.61; 95% CI, 0.44-0.84; *P* = .003 for OBS), and severe sepsis (OR, 0.36; 95% CI, 0.19-0.66; *P* < .001 for SAE; OR, 0.56; 95% CI, 0.36-0.88; *P* = .01 for OBS) in SAE and OBS was significantly lower than OS. In addition, the risk of ventilator-associated pneumonia (OR, 0.68; 95% CI, 0.49-0.95; *P* = .02) in SAE and the risks of acute kidney injury (OR, 0.70; 95% CI, 0.50-0.98; *P* = .04) and organ space surgical site infection (OR, 0.29; 95% CI, 0.12-0.72; *P* = .007) in OBS were significantly lower.

**Table 1.** Effect Sizes for SAE and OBS vs OS With Specific Complications Using Multivariable Logistic Regression and Inverse Probability Weighting

Outcome	No. of events/No. of patients	Multivariable logistic regression		Inverse probability weighting	
		OR (95% CI)	*P* value	OR (95% CI)	*P* value
AKI	271/12 930				
OS	120/3390	1 [Reference]		1 [Reference]	
SAE	52/2537	0.85 (0.59-1.23)	.39	0.88 (0.58-1.33)	.54
OBS	99/7003	0.70 (0.50-0.98)	.04	0.69 (0.48-0.98)	.04
ARDS	218/12930				
OS	109/3390	1 [Reference]		1 [Reference]	
SAE	26/2537	0.47 (0.30-0.75)	.001	0.36 (0.22-0.59)	<.001
OBS	83/7003	0.61 (0.43-0.86)	.006	0.57 (0.39-0.84)	.005
Cardiac arrest	269/12930				
OS	135/3390	1 [Reference]		1 [Reference]	
SAE	33/2537	0.53 (0.35-0.81)	.003	0.51 (0.31-0.83)	.007
OBS	101/7003	0.61 (0.44-0.84)	.003	0.59 (0.41-0.85)	.005
Deep SSI	34/12 930				
OS	14/3390	1 [Reference]		1 [Reference]	
SAE	7/2537	0.96 (0.36-2.58)	.93	1.39 (0.40-4.80)	.60
OBS	13/7003	0.63 (0.25-1.57)	.32	0.66 (0.29-1.53)	.34
Organ space SSI	42/12 930				
OS	25/3390	1 [Reference]		1 [Reference]	
SAE	7/2537	0.48 (0.20-1.20)	.12	0.43 (0.17-1.12)	.09
OBS	10/7003	0.29 (0.12-0.72)	.007	0.27 (0.12-0.64)	.003
Superficial SSI	40/12 930				
OS	15/3390	1 [Reference]		1 [Reference]	
SAE	4/2537	0.62 (0.19-2.01)	.43	0.81 (0.24-2.71)	.73
OBS	21/7003	1.30 (0.57-2.95)	.54	1.97 (0.56-6.88)	.29
Severe sepsis	140/12 930				
OS	70/3390	1 [Reference]		1 [Reference]	
SAE	14/2537	0.36 (0.19-0.66)	<.001	0.30 (0.15-0.58)	<.001
OBS	56/7003	0.56 (0.36-0.88)	.01	0.58 (0.34-0.97)	.0437
DVT	414/12 930				
OS	167/3390	1 [Reference]		1 [Reference]	
SAE	71/2537	0.83 (0.62-1.13)	.24	0.80 (0.55-1.15)	.23
OBS	176/7003	0.89 (0.68-1.16)	.39	0.84 (0.62-1.13)	.25
PE	227/12 930				
OS	84/3390	1 [Reference]		1 [Reference]	
SAE	34/2537	0.75 (0.49-1.15)	.19	0.65 (0.41-1.03)	.07
OBS	109/7003	0.97 (0.68-1.38)	.87	1.05 (0.72-1.51)	.81
Myocardial infarction	48/12 930				
OS	22/3390	1 [Reference]		1 [Reference]	
SAE	9/2537	0.75 (0.31-1.77)	.51	0.69 (0.30-1.58)	.38
OBS	17/7003	0.83 (0.37-1.85)	.65	0.76 (0.34-1.72)	.51
Stroke	143/12 930				
OS	61/3390	1 [Reference]		1 [Reference]	
SAE	22/2537	0.85 (0.50-1.45)	.55	0.81 (0.44-1.46)	.48
OBS	60/7003	0.91 (0.58-1.41)	.66	0.90 (0.53-1.53)	.71
Unplanned intubation	575/12930				
OS	189/3390	1 [Reference]		1 [Reference]	
SAE	118/2537	0.99 (0.76-1.28)	.93	0.92 (0.69-1.24)	.60
OBS	268/7003	0.89 (0.70-1.13)	.33	0.93 (0.71-1.22)	.60
Unplanned admission to ICU	537/12 930				
OS	146/3390	1 [Reference]		1 [Reference]	
SAE	103/2537	1.06 (0.80-1.40)	.67	1.02 (0.72-1.44)	.90
OBS	288/7003	1.22 (0.95-1.57)	.11	1.22 (0.89-1.67)	.22
Unplanned visit to operating room	482/12 930				
OS	192/3390	1 [Reference]		1 [Reference]	
SAE	86/2537	0.84 (0.64-1.12)	.23	0.69 (0.50-0.96)	.03
OBS	204/7003	0.87 (0.68-1.12)	.27	0.90 (0.65-1.25)	.53
CAUTI	76/12 930				
OS	29/3390	1 [Reference]		1 [Reference]	
SAE	16/2537	1.12 (0.57-2.19)	.75	0.84 (0.39-1.79)	.64
OBS	31/7003	0.72 (0.38-1.34)	.30	0.63 (0.31-1.29)	.21
VAP	426/12 930				
OS	193/3390	1 [Reference]		1 [Reference]	
SAE	53/2537	0.68 (0.49-0.95)	.02	0.54 (0.37-0.78)	.001
OBS	180/7003	0.96 (0.74-1.24)	.73	0.99 (0.70-1.39)	.94
Alcohol withdrawal syndrome	137/12930				
OS	34/3390	1 [Reference]		1 [Reference]	
SAE	32/2537	1.65 (0.95-2.87)	.07	1.60 (0.90-2.82)	.11
OBS	71/7003	1.47 (0.88-2.47)	.14	1.46 (0.88-2.42)	.14
Pressure ulcer	233/12 930				
OS	91/3390	1 [Reference]		1 [Reference]	
SAE	44/2537	0.98 (0.66-1.46)	.93	0.85 (0.55-1.32)	.48
OBS	98/7003	0.78 (0.55-1.11)	.16	0.77 (0.52-1.14)	.19

Abbreviations: AKI, acute kidney injury; ARDS, acute respiratory distress syndrome; CAUTI, catheter-associated urinary tract infection; DVT, deep vein thrombosis; ICU, intensive care unit; OBS, observation; OR, odds ratio; OS, open splenectomy; PE, pulmonary embolism; SAE, splenic angioembolization; SSI, surgical site infection; VAP, ventilator-associated pneumonia.

For other subgroups, there was significantly lower complication risk among SAE and OBS in shock index 1 or more, spleen AIS score 3 and spleen AIS score 4. In the subgroup spleen AIS score 5, the complication risk was the same (eTable 10 in Supplement 1).

### Multivariable Linear Regression Analysis for Hospital Course and Transfusion and Logistic Regression Analysis for Risk Factors of Failure

SAE (β, −1.37; 95% CI, −2.03 to −0.71; *P* < .001) and OBS (β, −1.33; 95% CI, −1.93 to −0.74; *P* < .001) showed a negative association with HLOS. SAE (β, −1.42; 95% CI, −1.87 to −0.96; *P* < .001) and OBS (β, −1.34, 95% CI, −1.75 to −0.92; *P* < .001) also showed a negative association with ICULOS (Table 2). The same trends were observed in normotension subgroup. However, in the hypotension subgroup, there were no significant associations between intervention groups and HLOS, ICULOS, or ventilator time (Table 2). In the failure subgroup, there were significant positive associations found between SAE and HLOS (β, 3.82; 95% CI, 1.67 to 5.97; *P* < .001), ICULOS (β, 2.82; 95% CI, 1.26 to 4.38; *P* < .001), and ventilator time (β, 2.10; 95% CI, 0.21 to 3.98; *P* = .03) (Table 2).

**Table 2.** Effect Sizes of SAE and OBS vs OS With Hospital Course Using Multivariable Linear Regression and Inverse Probability Weighting

Outcome	No. of patients	Mean (SD)	Multivariate linear regression		Inverse probability weighting	
			β (95% CI)	*P* value	β (95% CI)	*P* value
**Total**						
HLOS, d	12 930	13.75 (13.60)				
OS	3390	17.39 (15.48)	1 [Reference]		1 [Reference]	
SAE	2537	12.66 (11.34)	−1.37 (−2.03 to −0.71)	<.001	−2.53 (−3.41 to −1.64)	<.001
OBS	7003	12.38 (13.06)	−1.33 (−1.93 to −0.74)	<.001	−1.84 (−2.74 to −0.95)	<.001
ICULOS, d	11 083	7.97 (8.94)				
OS	3097	10.56 (10.58)	1 [Reference]		1 [Reference]	
SAE	2302	6.88 (8.16)	−1.42 (−1.87 to −0.96)	<.001	−2.26 (−2.92 to −1.61)	<.001
OBS	5684	7.00 (7.94)	−1.34 (−1.75 to −0.92)	<.001	−1.47 (−2.13 to −0.81)	<.001
Ventilator time, d	5436	7.81 (9.08)				
OS	2452	7.97 (9.34)	1 [Reference]		1 [Reference]	
SAE	832	7.36 (9.47)	−0.32 (−1.03 to 0.39)	.38	−0.23 (−1.09 to 0.64)	.61
OBS	2152	7.80 (8.60)	−0.04 (−0.62 to 0.55)	.90	0.78 (0.03 to 1.53)	.04
**SBP <90 mm Hg subgroup**						
HLOS, d	1483	18.64 (16.60)				
OS	751	19.15 (16.43)	1 [Reference]		1 [Reference]	
SAE	261	16.32 (11.85)	−1.60 (−3.93 to 0.74)	.18	−1.08 (−3.50 to 1.33)	.38
OBS	471	19.13 (18.90)	0.42 (−1.65 to 2.48)	.69	−0.03 (−2.30 to 2.24)	.98
ICULOS, d	1408	10.60 (9.90)				
OS	717	11.47 (10.14)	1 [Reference]		1 [Reference]	
SAE	251	8.51 (8.33)	−1.28 (−2.69 to 0.14)	.08	−0.89 (−2.72 to 0.94)	.34
OBS	440	10.37 (10.15)	0.07 (−1.18 to 1.32)	.91	−0.26 (−1.82 to 1.30)	.75
Ventilator time, d	1021	8.44 (9.50)				
OS	624	8.59 (9.83)	1 [Reference]		1 [Reference]	
SAE	128	7.47 (9.39)	−1.03 (−2.88 to 0.82)	.27	−1.47 (−3.14 to 0.20)	.09
OBS	269	8.58 (8.77)	0.14 (−1.37 to 1.65)	.86	0.82 (−0.77 to 2.40)	.31
**SBP ≥90 mm Hg subgroup**						
HLOS, d	11447	13.11 (13.03)				
OS	2639	16.89 (15.17)	1 [Reference]		1 [Reference]	
SAE	2276	12.24 (11.20)	−1.50 (−2.18 to −0.82)	<.001	−2.55 (−3.50 to −1.61)	<.001
OBS	6532	11.89 (12.39)	−1.53 (−2.15 to −0.92)	<.001	−1.99 (−2.93 to −1.05)	<.001
ICULOS, d	9675	7.59 (8.73)				
OS	2380	10.28 (10.69)	1 [Reference]		1 [Reference]	
SAE	2051	6.68 (8.12)	−1.49 (−1.97 to −1.00)	<.001	−2.34 (−3.06 to −1.61)	<.001
OBS	5244	6.72 (7.66)	−1.53 (−1.97 to −1.08)	<.001	−1.71 (−2.42 to −1.00)	<.001
Ventilator time, d	4415	7.66 (8.97)				
OS	1828	7.76 (9.17)	1 [Reference]		1 [Reference]	
SAE	704	7.34 (9.49)	−0.21 (−0.98 to 0.57)	.60	−0.19 (−1.13 to 0.75)	.69
OBS	1883	7.69 (8.57)	−0.04 (−0.67 to 0.60)	.91	0.73 (−0.07 to 1.53)	.07
**Failure subgroup**						
HLOS, d	4502	17.13 (15.21)				
OS	3390	17.39 (15.48)	1 [Reference]		1 [Reference]	
SAE	176	19.36 (14.41)	3.82 (1.67 to 5.97)	<.001	2.44 (−0.12 to 5.01)	.06
OBS	936	15.79 (14.27)	0.92 (−0.20 to 2.05)	.11	2.50 (−0.45 to 5.45)	.10
ICULOS, d	4080	10.24 (10.69)				
OS	3097	10.56 (10.58)	1 [Reference]		1 [Reference]	
SAE	168	11.65 (12.79)	2.82 (1.26 to 4.38)	<.001	1.64 (−0.31 to 3.58)	.10
OBS	815	8.72 (10.52)	0.08 (−0.77 to 0.92)	.86	0.76 (−1.66 to 3.18)	.54
Ventilator time, d	2914	8.05 (9.50)				
OS	2452	7.97 (9.34)	1 [Reference]		1 [Reference]	
SAE	98	9.21 (10.68)	2.10 (0.21 to 3.98)	.03	1.16 (−1.19 to 3.52)	.33
OBS	364	8.26 (10.16)	0.68 (−0.39 to 1.75)	.21	1.21 (−0.65 to 3.07)	.20

Abbreviations: HLOS, hospital length of stay; ICULOS, intensive care unit length of stay; OBS, observation; OS, open splenectomy; SAE, splenic angioembolization; SBP, systolic blood pressure.

SAE (β, −569; 95% CI, −610 to −529; *P* < .001) and OBS (β, −639; 95% CI, −676 to −603; *P* < .001) showed a negative association with packed red blood cell transfusion (eTable 11 in Supplement 1). Age, level of trauma center, SBP, heart rate, Glasgow Coma Scale score, alcohol use disorder, congestive heart failure, smoking, and spleen AIS score were independent risk factors for failure (eTable 12 in Supplement 1).

### Sensitivity Analysis

Consistent associations with mortality were observed across the 3 groups (HR, 0.66; 95% CI, 0.47-0.90; *P* = .01 for SAE and HR, 0.70; 95% CI, 0.55-0.89; *P* = .004 for OBS) (Figure 2C). In the subgroup analyses, the result of normotension subgroup was confirmed (Figure 2C). Sensitivity analysis confirmed that risk of any complication was significantly lower in SAE (OR, 0.69; 95% CI, 0.58-0.81; *P* < .001) and OBS (OR, 0.76; 95% CI, 0.66-0.88; *P* < .001) (Figure 2D). In the failure subgroup, the any complication risk increased significantly in SAE (OR, 2.19; 95% CI, 1.49-3.23; *P* < .001) and OBS (OR, 1.55; 95% CI, 1.15-2.07; *P* = .003) (Figure 2D). The risk of specific complications, such as ARDS, cardiac arrest, severe sepsis, acute kidney injury, organ space surgical site infection, and ventilator-associated pneumonia were also confirmed (Table 1). The effect size of SAE and OBS vs OS for HLOS, ICULOS, and ventilation days also received confirmation (Table 2).

Multivariable regression analyses were conducted to evaluate both primary and secondary outcomes at different temporal cutoffs (4 hours and 8 hours). The SAE and OBS cohorts demonstrated statistically superior outcomes compared with OS, including a significant reduction in mortality, lower incidence of complications, decreased rates of specific complications (ARDS, cardiac arrest, severe sepsis), and shorter hospital course (HLOS, ICULOS) (Table 3).

**Table 3.** Sensitivity Analysis of Outcomes Using Time Cutoffs

Outcome	4 h of Admission			8 h of Admission		
	No./total No. of patients	HR, OR, or β (95% CI)	*P* value	No./total No. of patients	HR, OR, or β (95% CI)	*P* value
**Mortality**						
OS	316/3070	1 [Reference]		339/3341	1 [Reference]	
SAE	59/1747	0.65 (0.48 to 0.87)	.004	85/2408	0.66 (0.51 to 0.85)	.001
OBS	311/8113	0.68 (0.56 to 0.82)	<.001	262/7181	0.62 (0.51 to 0.75)	<.001
**Any complications**						
OS	909/3070	1 [Reference]		975/3341	1 [Reference]	
SAE	297/1747	0.73 (0.62 to 0.86)	<.001	413/2408	0.73 (0.63 to 0.85)	<.001
OBS	1343/8113	0.78 (0.69 to 0.88)	<.001	1161/7181	0.76 (0.67 to 0.86)	<.001
**Specific complication**						
ARDS						
OS	103/3070	1 [Reference]		108/3341	1 [Reference]	
SAE	19/1747	0.49 (0.29 to 0.83)	.008	26/2408	0.50 (0.32 to 0.79)	.003
OBS	96/8113	0.57 (0.41 to 0.81)	.002	84/7181	0.59 (0.42 to 0.84)	.004
Cardiac arrest						
OS	125/3070	1 [Reference]		131/3341	1 [Reference]	
SAE	23/1747	0.57 (0.35 to 0.92)	.02	32/2408	0.57 (0.37 to 0.86)	.008
OBS	121/8113	0.62 (0.45 to 0.86)	.004	106/7181	0.64 (0.46 to 0.89)	.008
Severe sepsis						
OS	65/3070	1 [Reference]		70/3341	1 [Reference]	
SAE	8/1747	0.30 (0.14 to 0.64)	.002	13/2408	0.34 (0.18 to 0.64)	<.001
OBS	67/8113	0.58 (0.38 to 0.90)	.01	57/7181	0.54 (0.35 to 0.85)	.007
**Hospital course**						
HLOS, d	12 930			12 930		
OS	3070	1 [Reference]		3341	1 [Reference]	
SAE	1747	−1.31 (−2.05 to −0.57)	<.001	2408	−1.45 (−2.12 to −0.78)	<.001
OBS	8113	−1.24 (−1.84 to −0.64)	<.001	7181	−1.24 (−1.83 to −0.64)	<.001
ICULOS	11 083			11 083		
OS	2828	1 [Reference]		3054	1 [Reference]	
SAE	1607	−1.40 (−1.92 to −0.89)	<.001	2190	−1.39 (−1.86 to −0.93)	<.001
OBS	6648	−1.28 (−1.69 to −0.86)	<.001	5839	−1.28 (−1.70 to −0.87)	<.001

Abbreviations: ARDS, acute respiratory distress syndrome; HR, hazard ratio; HLOS, hospital length of stay; ICULOS, intensive care unit length of stay; OBS, observation; OR, odds ratio; OS, open splenectomy; SAE, splenic angioembolization.

## Discussion

This large-scale cohort study of 12 930 patients with multiple trauma and severe BSI demonstrates that splenic salvage strategies (SAE and OBS) are associated with significantly lower mortality, fewer complications, and shorter hospital stay compared with splenectomy. These findings align with evolving trauma paradigms favoring organ preservation^10^ and add to current evidence with the analysis of multitrauma populations, a cohort often excluded from prior studies.^3,11,12^

Splenic injuries remain a significant trauma burden. Given the spleen’s crucial role in immune function, efforts to preserve the organ when clinically feasible are advised.^13,14,15^ While nonoperative management achieves success in about 90% of BSI cases, hemodynamic instability and peritonitis remain indications for immediate splenectomy.^10^ Patients with multiple trauma and severe BSI represent a more common clinical scenario than isolated severe BSI.^12,16^ Concerns about concomitant injuries and secondary injury from hemorrhage after failure may lead surgeons to a lower threshold for operative intervention. In this study, the overall scope of injury and head injury was greater in the OS group. We conducted multivariate analyses to validate the findings regarding initial management. Adjustment for confounding factors reveals that NOM is associated with better outcomes. Also, splenectomy has been shown to increase mortality risk or is not independently associated with survival in patients with traumatic brain injury.^17,18,19^ Therefore, the multitrauma cases may present greater management challenges and therapeutic considerations.

In patients with multiple trauma and severe BSI, NOM presents a safe management strategy. The mortality benefit in our study persisted even after adjusting for injury severity and comorbidities, suggesting that splenic preservation may mitigate the cumulative physiologic insult of OS. Notably, patients who presented with hypotension had no increase in mortality and may benefit from the preservation of immune function in splenic salvage.^14,20,21^ But, a similar study from Japan showed that in-hospital mortality was the same.^22^ Also, comparable mortality was observed between OS and NOM patients in prior studies.^16,23,24^ This discrepancy may reflect evolving therapeutic protocols or case selection criteria, warranting investigation into potential confounding factors. While retrospective, our study is the only review to our knowledge exploring BSI in patients with multiple trauma. These results challenge the traditional treatment of emergent surgery in unstable patients and support recent reports advocating for SAE as a bridge to stability.^1,2^

The complication risk of NOM is lower, driven by reductions in severe sepsis, ARDS, and cardiac arrest. Our data support the findings from a recent meta-analysis that linked splenectomy to higher postoperative complications in high-grade injuries.^20^ These outcomes likely reflect the systemic inflammatory burden of splenectomy, which exacerbates posttraumatic immunosuppression and makes patients prone to infections.^14,16^ The spleen serves as a specialized lymphoid organ that filters blood-borne pathogens and coordinates humoral immunity. The spleen has distinctive architecture that enables phagocytosis of encapsulated organisms, particularly *Haemophilus influenzae*, *Streptococcus pneumoniae*, and *Neisseria meningitidis*, pathogens responsible for the life-threatening sepsis observed in asplenic patients.^21^ SAE patients had significantly higher pooled CD4+, CD4+/CD8+, and CD3+ cells.^20^ A prior large retrospective review of severe splenic injuries found that operative splenectomy was independently associated with early infectious complications beyond surgical site infection, including pneumonia, suggesting that the infection risk of splenectomy is likely in part due to immunologic changes rather than just surgical risk.^16^ The prospective multicenter study also showed splenectomy associated with higher infectious complications.^25^ In our analysis of patients with multiple trauma, chest is the most severely injured region outside of abdomen. The reduction in ARDS is particularly important for multitrauma patients, who often suffer concurrent thoracic injuries and whose overall injury burdens may present additive risk of ARDS development.

The safety of NOM in patients with multiple trauma and hypotension is a critical finding. Historically, a conservative approach was used for minor injuries with stable hemodynamics.^1^ Some small cohorts showed selective SAE served as a reliable therapeutic approach for hypotensive BSI.^5,26,27,28,29^ Previous TQIP studies have shown that SAE has emerged as an alternative treatment for isolated hypotensive BSI, with increasingly widespread adoption and comparable mortality and complication rates to OS.^3,4^ In another investigation of TQIP, including both isolated patients and patients with multiple trauma and hypotension, SAE had similar survival but shorter ICULOS compared with OS.^2^ A recent meta-analysis indicates that for hemodynamically unstable patients with blunt abdominal solid organ injuries who respond to fluid resuscitation, angioembolization was associated with high success rates, low mortality, and acceptable procedure-related complications.^5^ Our data suggest that SAE may mitigate the need for emergent laparotomy without compromising outcomes. The similar mortality rate, morbidity rate, and hospital course between NOM and OS in the hypotensive subgroup underscore the potential of angiography as a damage-control adjunct, particularly in centers with around-the-clock interventional radiology capabilities.

An important consideration is the consequence of treatment failure, especially in patients with multiple trauma. Our data mirror the 5.3% to 25% failure rates reported in recent cohorts, underscoring the importance of protocolized monitoring and early rescue interventions.^30,31^ Additionally, the mortality rate increased from 3.5% to 8.5% in SAE group and 3.6% to 4.5% in OBS group, although lower than the OS group. Failure of NOM was associated with significantly increased complications. The incidence of complications in the failure of SAE group was close to 50% and exceeded 30% in the failure of OBS group. These results emphasize the need for careful patient selection.

Our findings provide some practice thoughts. SAE may be considered as an alternative therapy, even in patients with hypotension. SAE may be used when transient hemodynamic stability is achievable with resuscitation. However, regional variability in SAE adoption persists, with rural centers often lacking angiography resources and fewer centers with all-hours access. A risk-stratified observation is that high-grade injuries in patients with multiple trauma warrant closer monitoring given the 13.4% failure rate in the OBS group. To prepare for treatment failure, institutions should establish rescue protocols because our SAE failure subgroup had a nearly 3-fold incidence of complications. We must exercise scientific prudence in extrapolating these observations; definitive establishment of splenic salvage criteria for multitrauma would necessitate randomized clinical trials.

### Limitations

Several constraints should be acknowledged. First, as with all large database analyses, our study may be susceptible to selection bias. While we used rigorous multivariable adjustments, key physiological parameters (eg, nadir SBP, peak heart rate) were unavailable in the dataset. The retrospective design also precluded assessment of patients’ hemodynamic responses to resuscitation before therapeutic interventions. Second, diagnostic certainty presents challenges in trauma populations. In hemodynamically unstable patients, definitive diagnosis of splenic injury is frequently established intraoperatively because of the absence of preoperative computed tomography imaging. Third, the American Association for the Surgery of Trauma guideline for grading splenic injury was changed in 2018, causing inconsistent diagnostic criteria.^32^ Fourth, our inclusion of patients with multiple trauma introduces complexity in outcome attribution. The composite nature of mortality and complication limits our ability to establish causality specifically related to splenic trauma. Furthermore, the analysis excluded laparoscopic and repair procedures because of insufficient sample sizes.

## Conclusions

This retrospective cohort study demonstrates that in patients with multiple trauma and severe BSI, both SAE and OBS were associated with favorable clinical outcomes compared with splenectomy, including reduced mortality, fewer complications (particularly ARDS, cardiac arrest, and severe sepsis), and shorter hospital and ICU stays. Notably, even among patients who were hypotensive on admission, nonoperative management (SAE or OBS) did not increase mortality or morbidity risks. However, cases in which there was failure of SAE/OBS showed elevated complication rates. These findings contribute to growing evidence supporting the safety of nonoperative approaches for high-grade splenic injuries, including in hemodynamically compromised patients. With appropriate patient selection criteria, spleen preservation strategies should be considered the preferred approach for severely injured patients with multiple trauma.

## Notes

Supplement 1. **eTable 1.** Adjusted variables in total cohort and subgroups **eTable 2.** The baseline of OS, SAE and OBS group **eTable 3.** Outcomes of OS, SAE and OBS groups **eTable 4.** The baseline of OS, SAE and OBS group (SBP < 90 mmHg) **eTable 5.** Outcomes of OS, SAE and OBS group (SBP<90 mmHg) **eTable 6.** The baseline of OS, SAE and OBS group (SBP ≥ 90 mmHg) **eTable 7.** Outcomes of OS, SAE and OBS group (SBP ≥ 90 mmHg) **eTable 8.** The baseline of OS, failure of SAE and failure of OBS group **eTable 9.** Outcomes of OS, failure of SAE and failure of OBS group **eTable 10.** Effect size of SAE and OBS vs OS with mortality and any complication of other subgroups **eTable 11.** Effect size of SAE and OBS vs OS with transfusion using multivariable linear regression analysis **eTable 12.** Univariable and multivariable logistic regression analysis for risk factors of failure **eFigure.** Patient selection flow diagram

Supplement 2. Data sharing statement
