# Supplementary material for: Severe Splenic Injuries in Patients With Multiple Trauma
Source: JAMA Surg. 2026 Feb 25;161(4):413–23. doi: 10.1001/jamasurg.2026.0016 (PMC12936967; doi:10.1001/jamasurg.2026.0016)
Supplement: Supplement 1. — eTable 1. Adjusted variables in total cohort and subgroups eTable 2. The baseline of OS, SAE and OBS group eTable 3. Outcomes of OS, SAE and OBS groups eTable 4. The baseline of OS, SAE and OBS group (SBP < 90 mmHg) eTable 5. Outcomes of OS, SAE and OBS group (SBP<90 mmHg) eTable 6. The baseline of OS, SAE and OBS group (SBP ≥ 90 mmHg) eTable 7. Outcomes of OS, SAE and OBS group (SBP ≥ 90 mmHg) eTable 8. The baseline of OS, failure of SAE and failure of OBS group eTable 9. Outcomes of OS, failure of SAE and failure of OBS group eTable 10. Effect size of SAE and OBS vs OS with mortality and any complication of other subgroups eTable 11. Effect size of SAE and OBS vs OS with transfusion using multivariable linear regression analysis eTable 12. Univariable and multivariable logistic regression analysis for risk factors of failure eFigure. Patient selection flow diagram [file jamasurg-e260016-s001.pdf]

Supplemental Online Content

Huang W, Braschi C, Jin F, Lewis M, Demetriades D. Severe splenic injuries in patients with multiple trauma. *JAMA Surg*. Published online February 25, 2026.
doi:10.1001/jamasurg.2026.0016

eTable 1. Adjusted variables in total cohort and subgroups
eTable 2. The baseline of OS, SAE and OBS group
eTable 3. Outcomes of OS, SAE and OBS groups
eTable 4. The baseline of OS, SAE and OBS group (SBP < 90 mmHg)
eTable 5. Outcomes of OS, SAE and OBS group (SBP<90 mmHg)
eTable 6. The baseline of OS, SAE and OBS group (SBP ≥ 90 mmHg)
eTable 7. Outcomes of OS, SAE and OBS group (SBP ≥ 90 mmHg)
eTable 8. The baseline of OS, failure of SAE and failure of OBS group
eTable 9. Outcomes of OS, failure of SAE and failure of OBS group
eTable 10. Effect size of SAE and OBS vs OS with mortality and any complication of other subgroups
eTable 11. Effect size of SAE and OBS vs OS with transfusion using multivariable linear regression analysis
eTable 12. Univariable and multivariable logistic regression analysis for risk factors of failure
eFigure. Patient selection flow diagram

This supplemental material has been provided by the authors to give readers additional information about their work.

© 2026 Huang W et al. *JAMA Surgery*.

eTable 1: adjusted variables in total cohort and subgroups	
Group	Adjusted variables
Total Cohort	age, sex, race, BMI, hospital type, payment, bed size, trauma level, SBP, HR, RR, GCS, temperature, pulse oximetry, respiratory assistance, alcohol use disorder, chemotherapy for cancer, congestive heart failure, smoking, chronic renal failure, diabetes mellitus, hypertension, steroid use, cirrhosis, anticoagulant therapy, mechanism, AIS of liver, spleen, kidney, pancreas, head, face, neck, chest, spine, upper extremity, lower extremity
Hypotension Subgroup: SBP < 90 mmHg	Age, BMI, bed size, trauma level, SBP, HR, GCS, temperature, pulse oximetry, respiratory assistance, diabetes mellitus, hypertension, COPD, steroid use, dementia, anticoagulant therapy, mental disorder, AIS of liver, spleen, kidney, pancreas, head, chest, spine, lower extremity
Normotension Subgroup: SBP ≥ 90 mmHg	age, sex, race, BMI, payment, bed size, trauma level, SBP, HR, RR, GCS, temperature, pulse oximetry, respiratory assistance, chemotherapy for cancer, congestive heart failure, smoking, chronic renal failure, diabetes mellitus, hypertension, steroid use, cirrhosis, anticoagulant therapy, substance abuse disorder, mechanism, AIS of liver, spleen, kidney, pancreas, head, face, neck, chest, spine, upper extremity, lower extremity
Failure	Age, race, hospital type, bed size, SBP, HR, RR, GCS, temperature, pulse oximetry, respiratory assistance, alcohol use disorder, bleeding disorder, congestive heart failure, smoking, cerebrovascular accident, diabetes mellitus, hypertension, COPD, steroid use, dementia, anticoagulant therapy, angina pectoris, mental disorder, mechanism, AIS of liver, spleen, kidney, pancreas, head, face, chest, spine, upper extremity, lower extremity

© 2026 Huang W et al. *JAMA Surgery.*

eTable 2: the baseline of OS, SAE and OBS group					
Variables	Total (n = 12930)	OS (n = 3390)	SAE (n = 2537)	OBS (n = 7003)	p
Age, Median (IQR)	39 (26, 56)	40 (27, 56)	42 (28, 58)	38 (26, 54)	< 0.001
≥55, n (%)	3477 (26.9)	944 (27.8)	794 (31.3)	1739 (24.8)	< 0.001
Sex, n (%)					
Male	9259 (71.6)	2483 (73.2)	1859 (73.3)	4917 (70.2)	< 0.001
Female	3671 (28.4)	907 (26.8)	678 (26.7)	2086 (29.8)	
Race, n (%)					
White	9864 (76.3)	2584 (76.2)	2003 (79.0)	5277 (75.4)	< 0.001
Black	1374 (10.6)	368 (10.9)	232 (9.1)	774 (11.1)	
Other	1362 (10.5)	328 (9.7)	248 (9.8)	786 (11.2)	
Unknow	330 (2.6)	110 (3.2)	54 (2.1)	166 (2.4)	
BMI, n (%)					
<30	8149 (63.0)	2224 (65.6)	1576 (62.1)	4349 (62.1)	< 0.001
≥30	3971 (30.7)	940 (27.7)	795 (31.3)	2236 (31.9)	
Unknow	810 (6.3)	226 (6.7)	166 (6.5)	418 (6.0)	
Hospital type, n (%)					
Non-Profit	11323 (87.6)	3010 (88.8)	2205 (86.9)	6108 (87.2)	0.138
Profit	1517 (11.7)	364 (10.7)	313 (12.3)	840 (12.0)	
Government	86 (0.7)	16 (0.5)	18 (0.7)	52 (0.7)	
Unknow	4 (0.0)	0 (0.0)	1 (0.0)	3 (0.0)	
Payment, n (%)					
Insurance	6485 (50.2)	1636 (48.3)	1316 (51.9)	3533 (50.4)	< 0.001
Medicaid	2453 (19.0)	663 (19.6)	443 (17.5)	1347 (19.2)	
Self-Pay	1593 (12.3)	454 (13.4)	290 (11.4)	849 (12.1)	
Medicare	1232 (9.5)	319 (9.4)	281 (11.1)	632 (9.0)	
Other	880 (6.8)	244 (7.2)	169 (6.7)	467 (6.7)	
Unknow	287 (2.2)	74 (2.2)	38 (1.5)	175 (2.5)	
Bed size, n (%)					
<200	686 (5.3)	198 (5.8)	158 (6.2)	330 (4.7)	0.003
201 to 400	3282 (25.4)	854 (25.2)	625 (24.6)	1803 (25.7)	
401 to 600	3844 (29.7)	998 (29.4)	704 (27.7)	2142 (30.6)	
More than 600	5118 (39.6)	1340 (39.5)	1050 (41.4)	2728 (39.0)	
Trauma Level, n (%)					
I	6732 (52.1)	1736 (51.2)	1327 (52.3)	3669 (52.4)	< 0.001
II	3440 (26.6)	839 (24.7)	641 (25.3)	1960 (28.0)	
III	28 (0.2)	8 (0.2)	2 (0.1)	18 (0.3)	
Unknow	2730 (21.1)	807 (23.8)	567 (22.3)	1356 (19.4)	
ED first vital signs					
SBP, Median (IQR)	121 (103, 139)	110 (90, 131)	121 (104, 138)	126 (110, 142)	< 0.001
<90, n (%)	1483 (11.5)	751 (22.2)	261 (10.3)	471 (6.7)	< 0.001

© 2026 Huang W et al. *JAMA Surgery.*

HR, Median (IQR)	97 (82, 114)	105 (86, 125)	95 (79, 111)	94 (81, 110)	< 0.001
≥120, n (%)	2560 (19.8)	1056 (31.2)	422 (16.6)	1082 (15.5)	< 0.001
RR, Median (IQR)	20 (18, 24)	20 (18, 25)	20 (18, 24)	20 (18, 23)	< 0.001
GCS, Median (IQR)	15 (13, 15)	14 (7, 15)	15 (14, 15)	15 (14, 15)	< 0.001
Shock Index, Median (Q1, Q3)	0.80 (0.64, 1.01)	0.95 (0.74, 1.23)	0.79 (0.63, 0.98)	0.75 (0.62, 0.92)	< 0.001
Temperature, n (%)					
<36℃	1606 (12.4)	559 (16.5)	309 (12.2)	738 (10.5)	< 0.001
≥36℃	9627 (74.5)	2156 (63.6)	1931 (76.1)	5540 (79.1)	
Unknow	1697 (13.1)	675 (19.9)	297 (11.7)	725 (10.4)	
Pulse oximetry, n (%)					
<90%	1061 (8.2)	411 (12.1)	171 (6.7)	479 (6.8)	< 0.001
≥90%	11693 (90.4)	2902 (85.6)	2339 (92.2)	6452 (92.1)	
Unknow	176 (1.4)	77 (2.3)	27 (1.1)	72 (1.0)	
Respiratory assistance, n (%)					
Yes	1731 (13.4)	745 (22.0)	239 (9.4)	747 (10.7)	< 0.001
No	10977 (84.9)	2600 (76.7)	2253 (88.8)	6124 (87.4)	
Unknow	222 (1.7)	45 (1.3)	45 (1.8)	132 (1.9)	
ISS, Median (IQR)	29 (22, 38)	38 (29, 43)	29 (26, 36)	27 (22, 34)	< 0.001
≥25, n (%)	9211 (71.2)	3134 (92.4)	2005 (79.0)	4072 (58.1)	< 0.001
Comorbidities, n (%)					
Alcohol Use Disorder	828 (6.4)	239 (7.1)	161 (6.3)	428 (6.1)	0.185
Bleeding Disorder	81 (0.6)	19 (0.6)	17 (0.7)	45 (0.6)	0.842
Chemotherapy for Cancer	18 (0.1)	3 (0.1)	7 (0.3)	8 (0.1)	0.134
Congestive Heart Failure	152 (1.2)	30 (0.9)	32 (1.3)	90 (1.3)	0.187
Smoking	3490 (27.0)	833 (24.6)	714 (28.1)	1943 (27.7)	0.001
Chronic Renal Failure	72 (0.6)	13 (0.4)	12 (0.5)	47 (0.7)	0.149
Cerebrovascular Accident	94 (0.7)	25 (0.7)	20 (0.8)	49 (0.7)	0.900
Diabetes Mellitus	1044 (8.1)	243 (7.2)	229 (9.0)	572 (8.2)	0.031
Hypertension	2425 (18.8)	599 (17.7)	533 (21.0)	1293 (18.5)	0.003
COPD	424 (3.3)	105 (3.1)	94 (3.7)	225 (3.2)	0.386
Steroid Use	68 (0.5)	15 (0.4)	21 (0.8)	32 (0.5)	0.064
Cirrhosis	189 (1.5)	61 (1.8)	47 (1.9)	81 (1.2)	0.007
Dementia	65 (0.5)	12 (0.4)	14 (0.6)	39 (0.6)	0.362
Anticoagulant Therapy	408 (3.2)	119 (3.5)	111 (4.4)	178 (2.5)	< 0.001
Angina Pectoris	4 (0.0)	0 (0.0)	0 (0.0)	4 (0.1)	0.320
Mental disorder	1236 (9.6)	301 (8.9)	258 (10.2)	677 (9.7)	0.223
Myocardial Infarction	50 (0.4)	17 (0.5)	6 (0.2)	27 (0.4)	0.266
Peripheral Arterial Disease	39 (0.3)	13 (0.4)	5 (0.2)	21 (0.3)	0.432
Substance Abuse Disorder	1662 (12.9)	448 (13.2)	337 (13.3)	877 (12.5)	0.473
Any comorbidities	7075 (54.7)	1801 (53.1)	1486 (58.6)	3788 (54.1)	< 0.001
Mechanism, n (%)					
MVT	10461 (80.9)	2864 (84.5)	1994 (78.6)	5603 (80.0)	< 0.001
Fall	1383 (10.7)	281 (8.3)	325 (12.8)	777 (11.1)	

© 2026 Huang W et al. *JAMA Surgery.*

Other	1086 (8.4)	245 (7.2)	218 (8.6)	623 (8.9)	
Abdominal solid organ AIS, n (%)					
Liver AIS=2	1775 (13.7)	698 (20.6)	276 (10.9)	801 (11.4)	< 0.001
Spleen					
AIS=3	6733 (52.1)	759 (22.4)	889 (35.0)	5085 (72.6)	< 0.001
AIS=4	3775 (29.2)	1183 (34.9)	1148 (45.3)	1444 (20.6)	
AIS=5	2422 (18.7)	1448 (42.7)	500 (19.7)	474 (6.8)	
Kidney AIS=2	1177 (9.1)	376 (11.1)	224 (8.8)	577 (8.2)	< 0.001
Pancreas AIS=2	331 (2.6)	230 (6.8)	22 (0.9)	79 (1.1)	< 0.001
AIS of each region, Median (IQR)					
Head	0 (0, 2)	1 (0, 3)	0 (0, 2)	0 (0, 2)	< 0.001
Face	0 (0, 1)	0 (0, 1)	0 (0, 1)	0 (0, 1)	0.004
Neck	0 (0, 0)	0 (0, 0)	0 (0, 0)	0 (0, 0)	< 0.001
Chest	3 (3, 3)	3 (3, 3)	3 (3, 3)	3 (3, 3)	< 0.001
Spine	0 (0, 2)	0 (0, 2)	0 (0, 2)	0 (0, 2)	< 0.001
Upper extremity	1 (0, 2)	1 (0, 2)	1 (0, 2)	1 (0, 2)	< 0.001
Lower extremity	1 (0, 3)	2 (0, 3)	1 (0, 2)	1 (0, 3)	< 0.001

BMI, Body Mass Index; SBP, systolic blood pressure; HR, heart rate; RR, respiratory rate; GCS, Glasgow Coma Scale; ISS, injury severity score; AIS, abbreviated injury score; ARDS, acute respiratory distress syndrome;

© 2026 Huang W et al. *JAMA Surgery*.

eTable 3: outcomes of OS, SAE and OBS groups					
Variables	Total (n = 12930)	OS (n = 3390)	SAE (n = 2537)	OBS (n = 7003)	p
Mortality, n (%)	686 (5.3)	344 (10.1)	88 (3.5)	254 (3.6)	< 0.001
Failure, n (%)	1134 (8.8)	22 (0.6)	176 (6.9)	936 (13.4)	< 0.001
Hospital course, Median (IQR), d					
HLOS	10 (6, 17)	13 (8, 22)	9 (6, 15)	9 (5, 15)	< 0.001
ICULOS	5 (3, 10)	7 (3, 14)	4 (3, 8)	4 (3, 8)	< 0.001
Ventilator time	4 (2, 10)	4 (2, 10)	4 (2, 9)	4 (2, 10)	0.050
Complications, n (%)					
AKI	271 (2.1)	120 (3.5)	52 (2.0)	99 (1.4)	< 0.001
ARDS	218 (1.7)	109 (3.2)	26 (1.0)	83 (1.2)	< 0.001
Cardiac Arrest	269 (2.1)	135 (4.0)	33 (1.3)	101 (1.4)	< 0.001
Deep SSI	34 (0.3)	14 (0.4)	7 (0.3)	13 (0.2)	0.104
Organ Space SSI	42 (0.3)	25 (0.7)	7 (0.3)	10 (0.1)	< 0.001
Superficial SSI	40 (0.3)	15 (0.4)	4 (0.2)	21 (0.3)	0.145
Severe Sepsis	140 (1.1)	70 (2.1)	14 (0.6)	56 (0.8)	< 0.001
VTE	590 (4.6)	230 (6.8)	99 (3.9)	261 (3.7)	< 0.001
DVT	414 (3.2)	167 (4.9)	71 (2.8)	176 (2.5)	< 0.001
PE	227 (1.8)	84 (2.5)	34 (1.3)	109 (1.6)	< 0.001
Myocardial Infarction	48 (0.4)	22 (0.6)	9 (0.4)	17 (0.2)	0.006
Stroke	143 (1.1)	61 (1.8)	22 (0.9)	60 (0.9)	< 0.001
Unplanned Intubation	575 (4.4)	189 (5.6)	118 (4.7)	268 (3.8)	< 0.001
Unplanned Admission to ICU	537 (4.2)	146 (4.3)	103 (4.1)	288 (4.1)	0.867
Unplanned Visit to OR	482 (3.7)	192 (5.7)	86 (3.4)	204 (2.9)	< 0.001
CAUTI	76 (0.6)	29 (0.9)	16 (0.6)	31 (0.4)	0.034
CLABI	32 (0.2)	16 (0.5)	1 (0.0)	15 (0.2)	0.003
VAP	426 (3.3)	193 (5.7)	53 (2.1)	180 (2.6)	< 0.001
Alcohol Withdrawal Syndrome	137 (1.1)	34 (1.0)	32 (1.3)	71 (1.0)	0.541
Pressure Ulcer	233 (1.8)	91 (2.7)	44 (1.7)	98 (1.4)	< 0.001
Any Complications	2549 (19.7)	989 (29.2)	441 (17.4)	1119 (16.0)	< 0.001
Transfusion, Mean (SD), ml					
PRBC	449 (888)	1110 (1233)	310 (608)	181 (546)	< 0.001
Plasma	339 (714)	743 (963)	178 (438)	135 (445)	< 0.001
Platelet	54 (138)	123 (189)	30 (101)	19 (85)	< 0.001

HLOS, hospital length of stay; ICULOS, intensive care unit length of stay; AKI, acute kidney injury; ARDS, acute respiratory distress syndrome; SSI, surgical site infection; VTE: venous thromboembolism; DVT, deep vein thrombosis; PE, pulmonary embolism; VAP, ventilator associated pneumonia; CAUTI: catheter associated urinary tract infection; CLABI: central line associated bloodstream infection; OR, operating room; PRBC, packed red blood cell.

© 2026 Huang W et al. *JAMA Surgery*.

eTable 4: the baseline of OS, SAE and OBS group (SBP < 90 mmHg)					
Variables	Total (n = 1483)	OS (n = 751)	SAE (n = 261)	OBS (n = 471)	p
Age, Median (IQR)	44 (28, 59)	44 (28, 59)	48 (29, 62)	42 (29, 56)	0.022
≥55, n (%)	484 (32.6)	251 (33.4)	106 (40.6)	127 (27.0)	< 0.001
Sex, n (%)					
Male	1011 (68.2)	527 (70.2)	176 (67.4)	308 (65.4)	0.209
Female	472 (31.8)	224 (29.8)	85 (32.6)	163 (34.6)	
Race, n (%)					
White	1161 (78.3)	593 (79.0)	206 (78.9)	362 (76.9)	0.440
Black	149 (10.0)	81 (10.8)	20 (7.7)	48 (10.2)	
Other	130 (8.8)	59 (7.9)	24 (9.2)	47 (10.0)	
Unknow	43 (2.9)	18 (2.4)	11 (4.2)	14 (3.0)	
BMI, n (%)					
<30	935 (63.0)	500 (66.6)	152 (58.2)	283 (60.1)	0.057
≥30	465 (31.4)	210 (28.0)	95 (36.4)	160 (34.0)	
Unknow	83 (5.6)	41 (5.5)	14 (5.4)	28 (5.9)	
Hospital type, n (%)					
Non-Profit	1293 (87.2)	663 (88.3)	227 (87.0)	403 (85.6)	0.665
Profit	182 (12.3)	85 (11.3)	33 (12.6)	64 (13.6)	
Government	7 (0.5)	3 (0.4)	1 (0.4)	3 (0.6)	
Unknow	1 (0.1)	0 (0.0)	0 (0.0)	1 (0.2)	
Payment, n (%)					
Insurance	727 (49.0)	368 (49.0)	129 (49.4)	230 (48.8)	0.656
Medicaid	277 (18.7)	139 (18.5)	45 (17.2)	93 (19.7)	
Self-Pay	176 (11.9)	94 (12.5)	30 (11.5)	52 (11.0)	
Medicare	172 (11.6)	86 (11.5)	38 (14.6)	48 (10.2)	
Other	96 (6.5)	50 (6.7)	14 (5.4)	32 (6.8)	
Unknow	35 (2.4)	14 (1.9)	5 (1.9)	16 (3.4)	
Bed size, n (%)					
<200	89 (6.0)	49 (6.5)	16 (6.1)	24 (5.1)	0.056
201 to 400	341 (23.0)	163 (21.7)	58 (22.2)	120 (25.5)	
401 to 600	434 (29.3)	229 (30.5)	59 (22.6)	146 (31.0)	
More than 600	619 (41.7)	310 (41.3)	128 (49.0)	181 (38.4)	
Trauma Level, n (%)					
I	779 (52.5)	394 (52.5)	137 (52.5)	248 (52.7)	0.150
II	392 (26.4)	185 (24.6)	66 (25.3)	141 (29.9)	
III	2 (0.1)	1 (0.1)	0 (0.0)	1 (0.2)	
Unknow	310 (20.9)	171 (22.8)	58 (22.2)	81 (17.2)	
ED first vital signs					
SBP, Median (IQR)	78 (70, 84)	78 (68, 82)	80 (71, 85)	79 (70, 84)	< 0.001
HR, Median (IQR)	104 (84, 124)	109 (88, 128)	97 (78, 115)	100 (83, 123)	< 0.001
≥120, n (%)	455 (30.7)	267 (35.6)	49 (18.8)	139 (29.5)	< 0.001

© 2026 Huang W et al. *JAMA Surgery*.

RR, Median (IQR)	20 (17, 25)	20 (17, 26)	20 (18, 24)	20 (16, 25)	0.283
GCS, Median (IQR)	14 (4, 15)	14 (3, 15)	15 (14, 15)	14 (6, 15)	< 0.001
Shock Index, Median (Q1, Q3)	1.36 (1.10, 1.68)	1.42 (1.16, 1.76)	1.23 (1.00, 1.50)	1.35 (1.07, 1.63)	< 0.001
Temperature, n (%)					
<36℃	277 (18.7)	145 (19.3)	42 (16.1)	90 (19.1)	< 0.001
≥36℃	897 (60.5)	412 (54.9)	177 (67.8)	308 (65.4)	
Unknow	309 (20.8)	194 (25.8)	42 (16.1)	73 (15.5)	
Pulse oximetry, n (%)					
<90%	201 (13.6)	113 (15.0)	33 (12.6)	55 (11.7)	0.105
≥90%	1246 (84.0)	614 (81.8)	225 (86.2)	407 (86.4)	
Unknow	36 (2.4)	24 (3.2)	3 (1.1)	9 (1.9)	
Respiratory assistance, n (%)					
Yes	382 (25.8)	228 (30.4)	39 (14.9)	115 (24.4)	< 0.001
No	1080 (72.8)	513 (68.3)	220 (84.3)	347 (73.7)	
Unknow	21 (1.4)	10 (1.3)	2 (0.8)	9 (1.9)	
ISS, Median (IQR)	34 (27, 43)	38 (29, 45)	34 (27, 38)	29 (22, 38)	< 0.001
≥25, n (%)	1269 (85.6)	702 (93.5)	220 (84.3)	347 (73.7)	< 0.001
Comorbidities, n (%)					
Alcohol Use Disorder	131 (8.8)	63 (8.4)	22 (8.4)	46 (9.8)	0.689
Bleeding Disorder	11 (0.7)	6 (0.8)	2 (0.8)	3 (0.6)	1.000
Chemotherapy for Cancer	1 (0.1)	1 (0.1)	0 (0.0)	0 (0.0)	1.000
Congestive Heart Failure	18 (1.2)	10 (1.3)	4 (1.5)	4 (0.8)	0.639
Smoking	368 (24.8)	176 (23.4)	72 (27.6)	120 (25.5)	0.377
Chronic Renal Failure	12 (0.8)	5 (0.7)	1 (0.4)	6 (1.3)	0.463
Cerebrovascular Accident	13 (0.9)	4 (0.5)	4 (1.5)	5 (1.1)	0.243
Diabetes Mellitus	130 (8.8)	56 (7.5)	33 (12.6)	41 (8.7)	0.038
Hypertension	296 (20.0)	142 (18.9)	68 (26.1)	86 (18.3)	0.024
COPD	52 (3.5)	23 (3.1)	16 (6.1)	13 (2.8)	0.038
Steroid Use	10 (0.7)	5 (0.7)	4 (1.5)	1 (0.2)	0.106
Cirrhosis	44 (3.0)	24 (3.2)	10 (3.8)	10 (2.1)	0.372
Dementia	10 (0.7)	2 (0.3)	3 (1.1)	5 (1.1)	0.097
Anticoagulant Therapy	70 (4.7)	37 (4.9)	21 (8.0)	12 (2.5)	0.003
Angina Pectoris	0 (0.0)	0 (0.0)	0 (0.0)	0 (0.0)	1.000
Mental disorder	144 (9.7)	60 (8.0)	29 (11.1)	55 (11.7)	0.074
Myocardial Infarction	9 (0.6)	7 (0.9)	0 (0.0)	2 (0.4)	0.258
Peripheral Arterial Disease	4 (0.3)	3 (0.4)	0 (0.0)	1 (0.2)	0.827
Substance Abuse Disorder	188 (12.7)	84 (11.2)	36 (13.8)	68 (14.4)	0.210
Any comorbidities	835 (56.3)	391 (52.1)	171 (65.5)	273 (58.0)	< 0.001
Mechanism, n (%)					
MVT	1215 (81.9)	619 (82.4)	209 (80.1)	387 (82.2)	0.302
Fall	148 (10.0)	69 (9.2)	35 (13.4)	44 (9.3)	
Other	120 (8.1)	63 (8.4)	17 (6.5)	40 (8.5)	
Abdominal solid organ AIS, n (%)					

© 2026 Huang W et al. *JAMA Surgery*.

Liver AIS=2	282 (19.0)	173 (23.0)	30 (11.5)	79 (16.8)	< 0.001
Spleen					
AIS=3	589 (39.7)	170 (22.6)	92 (35.2)	327 (69.4)	< 0.001
AIS=4	429 (28.9)	225 (30.0)	112 (42.9)	92 (19.5)	
AIS=5	465 (31.4)	356 (47.4)	57 (21.8)	52 (11.0)	
Kidney AIS=2	189 (12.7)	107 (14.2)	32 (12.3)	50 (10.6)	0.174
Pancreas AIS=2	74 (5.0)	61 (8.1)	3 (1.1)	10 (2.1)	< 0.001
AIS of each region, Median (IQR)					
Head	1 (0, 3)	1 (0, 3)	1 (0, 2)	1 (0, 3)	< 0.001
Face	0 (0, 1)	0 (0, 1)	0 (0, 1)	0 (0, 1)	0.589
Neck	0 (0, 0)	0 (0, 0)	0 (0, 0)	0 (0, 0)	0.228
Chest	3 (3, 3)	3 (3, 4)	3 (3, 3)	3 (3, 3)	0.003
Spine	2 (0, 2)	2 (0, 2)	1 (0, 2)	2 (0, 2)	0.189
Upper extremity	1 (0, 2)	1 (0, 2)	1 (0, 2)	1 (0, 2)	0.922
Lower extremity	2 (0, 3)	2 (0, 3)	2 (0, 3)	2 (0, 3)	0.053

BMI, Body Mass Index; SBP, systolic blood pressure; HR, heart rate; RR, respiratory rate; GCS, Glasgow Coma Scale; ISS, injury severity score; AIS, abbreviated injury score; ARDS, acute respiratory distress syndrome;

© 2026 Huang W et al. *JAMA Surgery*.

eTable 5: outcomes of OS, SAE and OBS group (SBP<90 mmHg)					
Variables	Total (n = 1483)	OS (n = 751)	SAE (n = 261)	OBS (n = 471)	p
Mortality, n (%)	174 (11.7)	114 (15.2)	16 (6.1)	44 (9.3)	< 0.001
Failure, n (%)	100 (6.7)	5 (0.7)	29 (11.1)	66 (14.0)	< 0.001
Hospital course, Median (IQR), d					
HLOS	14 (8, 23)	14 (8, 25)	13 (8, 20)	14 (7, 24)	0.264
ICULOS	7 (4, 15)	8 (4, 16)	6 (3, 11)	6 (3, 14)	< 0.001
Ventilator time	5 (2, 11)	5 (2, 11)	4 (2, 8)	6 (3, 11)	0.485
Complications, n (%)					
AKI	58 (3.9)	33 (4.4)	7 (2.7)	18 (3.8)	0.466
ARDS	37 (2.5)	28 (3.7)	2 (0.8)	7 (1.5)	0.007
Cardiac Arrest	51 (3.4)	34 (4.5)	5 (1.9)	12 (2.5)	0.060
Deep SSI	10 (0.7)	3 (0.4)	0 (0.0)	7 (1.5)	0.028
Organ Space SSI	10 (0.7)	6 (0.8)	1 (0.4)	3 (0.6)	0.912
Superficial SSI	10 (0.7)	4 (0.5)	2 (0.8)	4 (0.8)	0.687
Severe Sepsis	32 (2.2)	20 (2.7)	2 (0.8)	10 (2.1)	0.192
VTE	114 (7.7)	60 (8.0)	15 (5.7)	39 (8.3)	0.424
DVT	83 (5.6)	44 (5.9)	11 (4.2)	28 (5.9)	0.563
PE	38 (2.6)	19 (2.5)	5 (1.9)	14 (3.0)	0.685
Myocardial Infarction	11 (0.7)	8 (1.1)	2 (0.8)	1 (0.2)	0.227
Stroke	38 (2.6)	20 (2.7)	4 (1.5)	14 (3.0)	0.483
Unplanned Intubation	96 (6.5)	43 (5.7)	20 (7.7)	33 (7.0)	0.467
Unplanned Admission to ICU	80 (5.4)	34 (4.5)	15 (5.7)	31 (6.6)	0.291
Unplanned Visit to OR	87 (5.9)	49 (6.5)	12 (4.6)	26 (5.5)	0.484
CAUTI	15 (1.0)	11 (1.5)	2 (0.8)	2 (0.4)	0.209
CLABI	9 (0.6)	6 (0.8)	0 (0.0)	3 (0.6)	0.489
VAP	98 (6.6)	63 (8.4)	10 (3.8)	25 (5.3)	0.015
Alcohol Withdrawal Syndrome	22 (1.5)	12 (1.6)	2 (0.8)	8 (1.7)	0.662
Pressure Ulcer	54 (3.6)	24 (3.2)	10 (3.8)	20 (4.2)	0.624
Any Complications	475 (32.0)	268 (35.7)	67 (25.7)	140 (29.7)	0.005
Transfusion, Mean (SD), ml					
PRBC	1155 (1308)	1521 (1406)	817 (987)	759 (1126)	< 0.001
Plasma	774 (1000)	1007 (1049)	482 (744)	513 (927)	< 0.001
Platelet	130 (194)	180 (208)	77 (161)	68 (158)	< 0.001

HLOS, hospital length of stay; ICULOS, intensive care unit length of stay; AKI, acute kidney injury; ARDS, acute respiratory distress syndrome; SSI, surgical site infection; VTE: venous thromboembolism; DVT, deep vein thrombosis; PE, pulmonary embolism; VAP, ventilator associated pneumonia; CAUTI: catheter associated urinary tract infection; CLABI: central line associated bloodstream infection; OR, operating room; PRBC, packed red blood cell.

© 2026 Huang W et al. *JAMA Surgery.*

eTable 6: the baseline of OS, SAE and OBS group (SBP ≥ 90 mmHg)					
Variables	Total (n = 11447)	OS (n = 2639)	SAE (n = 2276)	OBS (n = 6532)	p
Age, Median (IQR)	39 (26, 55)	39 (27, 55)	42 (28, 57)	37 (25, 54)	< 0.001
≥55, n (%)	2993 (26.1)	693 (26.3)	688 (30.2)	1612 (24.7)	< 0.001
Sex, n (%)					
Male	8248 (72.1)	1956 (74.1)	1683 (73.9)	4609 (70.6)	< 0.001
Female	3199 (27.9)	683 (25.9)	593 (26.1)	1923 (29.4)	
Race, n (%)					
White	8703 (76.0)	1991 (75.4)	1797 (79.0)	4915 (75.2)	< 0.001
Black	1225 (10.7)	287 (10.9)	212 (9.3)	726 (11.1)	
Other	1232 (10.8)	269 (10.2)	224 (9.8)	739 (11.3)	
Unknow	287 (2.5)	92 (3.5)	43 (1.9)	152 (2.3)	
BMI, n (%)					
<30	7214 (63.0)	1724 (65.3)	1424 (62.6)	4066 (62.2)	0.002
≥30	3506 (30.6)	730 (27.7)	700 (30.8)	2076 (31.8)	
Unknow	727 (6.4)	185 (7.0)	152 (6.7)	390 (6.0)	
Hospital type, n (%)					
Non-Profit	10030 (87.6)	2347 (88.9)	1978 (86.9)	5705 (87.3)	0.234
Profit	1335 (11.7)	279 (10.6)	280 (12.3)	776 (11.9)	
Government	79 (0.7)	13 (0.5)	17 (0.7)	49 (0.8)	
Unknow	3 (0.0)	0 (0.0)	1 (0.0)	2 (0.0)	
Payment, n (%)					
Insurance	5758 (50.3)	1268 (48.0)	1187 (52.2)	3303 (50.6)	0.002
Medicaid	2176 (19.0)	524 (19.9)	398 (17.5)	1254 (19.2)	
Self-Pay	1417 (12.4)	360 (13.6)	260 (11.4)	797 (12.2)	
Medicare	1060 (9.3)	233 (8.8)	243 (10.7)	584 (8.9)	
Other	784 (6.8)	194 (7.4)	155 (6.8)	435 (6.7)	
Unknow	252 (2.2)	60 (2.3)	33 (1.4)	159 (2.4)	
Bed size, n (%)					
<200	597 (5.2)	149 (5.6)	142 (6.2)	306 (4.7)	0.028
201 to 400	2941 (25.7)	691 (26.2)	567 (24.9)	1683 (25.8)	
401 to 600	3410 (29.8)	769 (29.1)	645 (28.3)	1996 (30.6)	
More than 600	4499 (39.3)	1030 (39.0)	922 (40.5)	2547 (39.0)	
Trauma Level, n (%)					
I	5953 (52.0)	1342 (50.9)	1190 (52.3)	3421 (52.4)	< 0.001
II	3048 (26.6)	654 (24.8)	575 (25.3)	1819 (27.8)	
III	26 (0.2)	7 (0.3)	2 (0.1)	17 (0.3)	
Unknow	2420 (21.1)	636 (24.1)	509 (22.4)	1275 (19.5)	
ED first vital signs					
SBP, Median (IQR)	125 (110, 141)	118 (104, 137)	124 (110, 139)	128 (113, 143)	< 0.001
HR, Median (IQR)	96 (82, 113)	104 (86, 124)	95 (80, 111)	94 (81, 110)	< 0.001
≥120, n (%)	2105 (18.4)	789 (29.9)	373 (16.4)	943 (14.4)	< 0.001

© 2026 Huang W et al. *JAMA Surgery.*

RR, Median (IQR)	20 (18, 24)	20 (18, 24)	20 (18, 24)	20 (18, 23)	< 0.001
GCS, Median (IQR)	15 (14, 15)	14 (8, 15)	15 (14, 15)	15 (14, 15)	< 0.001
Shock Index, Median (Q1, Q3)	0.76 (0.62, 0.93)	0.86 (0.70, 1.07)	0.76 (0.62, 0.92)	0.73 (0.61, 0.88)	< 0.001
Temperature, n (%)					
<36℃	1329 (11.6)	414 (15.7)	267 (11.7)	648 (9.9)	< 0.001
≥36℃	8730 (76.3)	1744 (66.1)	1754 (77.1)	5232 (80.1)	
Unknow	1388 (12.1)	481 (18.2)	255 (11.2)	652 (10.0)	
Pulse oximetry, n (%)					
<90%	860 (7.5)	298 (11.3)	138 (6.1)	424 (6.5)	< 0.001
≥90%	10447 (91.3)	2288 (86.7)	2114 (92.9)	6045 (92.5)	
Unknow	140 (1.2)	53 (2.0)	24 (1.1)	63 (1.0)	
Respiratory assistance, n (%)					
Yes	1349 (11.8)	517 (19.6)	200 (8.8)	632 (9.7)	< 0.001
No	9897 (86.5)	2087 (79.1)	2033 (89.3)	5777 (88.4)	
Unknow	201 (1.8)	35 (1.3)	43 (1.9)	123 (1.9)	
ISS, Median (IQR)	29 (22, 36)	36 (29, 43)	29 (25, 36)	27 (22, 34)	< 0.001
≥25, n (%)	7942 (69.4)	2432 (92.2)	1785 (78.4)	3725 (57.0)	< 0.001
Comorbidities, n (%)					
Alcohol Use Disorder	697 (6.1)	176 (6.7)	139 (6.1)	382 (5.8)	0.330
Bleeding Disorder	70 (0.6)	13 (0.5)	15 (0.7)	42 (0.6)	0.669
Chemotherapy for Cancer	17 (0.1)	2 (0.1)	7 (0.3)	8 (0.1)	0.090
Congestive Heart Failure	134 (1.2)	20 (0.8)	28 (1.2)	86 (1.3)	0.076
Smoking	3122 (27.3)	657 (24.9)	642 (28.2)	1823 (27.9)	0.007
Chronic Renal Failure	60 (0.5)	8 (0.3)	11 (0.5)	41 (0.6)	0.143
Cerebrovascular Accident	81 (0.7)	21 (0.8)	16 (0.7)	44 (0.7)	0.819
Diabetes Mellitus	914 (8.0)	187 (7.1)	196 (8.6)	531 (8.1)	0.116
Hypertension	2129 (18.6)	457 (17.3)	465 (20.4)	1207 (18.5)	0.019
COPD	372 (3.2)	82 (3.1)	78 (3.4)	212 (3.2)	0.819
Steroid Use	58 (0.5)	10 (0.4)	17 (0.7)	31 (0.5)	0.166
Cirrhosis	145 (1.3)	37 (1.4)	37 (1.6)	71 (1.1)	0.110
Dementia	55 (0.5)	10 (0.4)	11 (0.5)	34 (0.5)	0.674
Anticoagulant Therapy	338 (3.0)	82 (3.1)	90 (4.0)	166 (2.5)	0.002
Angina Pectoris	4 (0.0)	0 (0.0)	0 (0.0)	4 (0.1)	0.506
Mental disorder	1092 (9.5)	241 (9.1)	229 (10.1)	622 (9.5)	0.541
Myocardial Infarction	41 (0.4)	10 (0.4)	6 (0.3)	25 (0.4)	0.700
Peripheral Arterial Disease	35 (0.3)	10 (0.4)	5 (0.2)	20 (0.3)	0.601
Substance Abuse Disorder	1474 (12.9)	364 (13.8)	301 (13.2)	809 (12.4)	0.163

© 2026 Huang W et al. *JAMA Surgery*.

Any comorbidities	6240 (54.5)	1410 (53.4)	1315 (57.8)	3515 (53.8)	0.002
Mechanism, n (%)					
MVT	9246 (80.8)	2245 (85.1)	1785 (78.4)	5216 (79.9)	< 0.001
Fall	1235 (10.8)	212 (8.0)	290 (12.7)	733 (11.2)	
Other	966 (8.4)	182 (6.9)	201 (8.8)	583 (8.9)	
Abdominal solid organ AIS, n (%)					
Liver AIS=2	1493 (13.0)	525 (19.9)	246 (10.8)	722 (11.1)	< 0.001
Spleen					
AIS=3	6144 (53.7)	589 (22.3)	797 (35.0)	4758 (72.8)	< 0.001
AIS=4	3346 (29.2)	958 (36.3)	1036 (45.5)	1352 (20.7)	
AIS=5	1957 (17.1)	1092 (41.4)	443 (19.5)	422 (6.5)	
Kidney AIS=2	988 (8.6)	269 (10.2)	192 (8.4)	527 (8.1)	0.004
Pancreas AIS=2	257 (2.2)	169 (6.4)	19 (0.8)	69 (1.1)	< 0.001
AIS of each region, Median (IQR)					
Head	0 (0, 2)	1 (0, 3)	0 (0, 2)	0 (0, 2)	< 0.001
Face	0 (0, 1)	0 (0, 1)	0 (0, 1)	0 (0, 1)	0.012
Neck	0 (0, 0)	0 (0, 0)	0 (0, 0)	0 (0, 0)	< 0.001
Chest	3 (3, 3)	3 (3, 3)	3 (3, 3)	3 (3, 3)	< 0.001
Spine	0 (0, 2)	0 (0, 2)	0 (0, 2)	0 (0, 2)	< 0.001
Upper extremity	1 (0, 2)	1 (0, 2)	1 (0, 2)	1 (0, 2)	< 0.001
Lower extremity	1 (0, 3)	1 (0, 3)	1 (0, 2)	1 (0, 3)	< 0.001

BMI, Body Mass Index; SBP, systolic blood pressure; HR, heart rate; RR, respiratory rate; GCS, Glasgow Coma Scale; ISS, injury severity score; AIS, abbreviated injury score; ARDS, acute respiratory distress syndrome;

© 2026 Huang W et al. *JAMA Surgery*.

eTable 7: outcomes of OS, SAE and OBS group (SBP ≥ 90 mmHg)					
Variables	Total (n = 11447)	OS (n = 2639)	SAE (n = 2276)	OBS (n = 6532)	p
Mortality, n (%)	512 (4.5)	230 (8.7)	72 (3.2)	210 (3.2)	< 0.001
Failure, n (%)	1034 (9.0)	17 (0.6)	147 (6.5)	870 (13.3)	< 0.001
Hospital course, Median (IQR), d					
HLOS	9 (6, 16)	12 (7, 21)	9 (6, 14)	8 (5, 14)	< 0.001
ICULOS	4 (3, 9)	6 (3, 13)	4 (3, 7)	4 (3, 7)	< 0.001
Ventilator time	4 (2, 10)	4 (2, 10)	4 (2, 9)	4 (2, 10)	0.093
Complications, n (%)					
AKI	213 (1.9)	87 (3.3)	45 (2.0)	81 (1.2)	< 0.001
ARDS	181 (1.6)	81 (3.1)	24 (1.1)	76 (1.2)	< 0.001
Cardiac Arrest	218 (1.9)	101 (3.8)	28 (1.2)	89 (1.4)	< 0.001
Deep SSI	24 (0.2)	11 (0.4)	7 (0.3)	6 (0.1)	0.002
Organ Space SSI	32 (0.3)	19 (0.7)	6 (0.3)	7 (0.1)	< 0.001
Superficial SSI	30 (0.3)	11 (0.4)	2 (0.1)	17 (0.3)	0.080
Severe Sepsis	108 (0.9)	50 (1.9)	12 (0.5)	46 (0.7)	< 0.001
VTE	476 (4.2)	170 (6.4)	84 (3.7)	222 (3.4)	< 0.001
DVT	331 (2.9)	123 (4.7)	60 (2.6)	148 (2.3)	< 0.001
PE	189 (1.7)	65 (2.5)	29 (1.3)	95 (1.5)	< 0.001
Myocardial Infarction	37 (0.3)	14 (0.5)	7 (0.3)	16 (0.2)	0.092
Stroke	105 (0.9)	41 (1.6)	18 (0.8)	46 (0.7)	< 0.001
Unplanned Intubation	479 (4.2)	146 (5.5)	98 (4.3)	235 (3.6)	< 0.001
Unplanned Admission to ICU	457 (4.0)	112 (4.2)	88 (3.9)	257 (3.9)	0.746
Unplanned Visit to OR	395 (3.5)	143 (5.4)	74 (3.3)	178 (2.7)	< 0.001
CAUTI	61 (0.5)	18 (0.7)	14 (0.6)	29 (0.4)	0.305
CLABI	23 (0.2)	10 (0.4)	1 (0.0)	12 (0.2)	0.033
VAP	328 (2.9)	130 (4.9)	43 (1.9)	155 (2.4)	< 0.001
Alcohol Withdrawal Syndrome	115 (1.0)	22 (0.8)	30 (1.3)	63 (1.0)	0.209
Pressure Ulcer	179 (1.6)	67 (2.5)	34 (1.5)	78 (1.2)	< 0.001
Any Complications	2074 (18.1)	721 (27.3)	374 (16.4)	979 (15.0)	< 0.001
Transfusion, Mean (SD), ml					
PRBC	358 (773)	994 (1153)	252 (518)	138 (450)	< 0.001
Plasma	267 (626)	661 (920)	137 (359)	98 (347)	< 0.001
Platelet	42 (123)	106 (179)	23 (88)	14 (73)	< 0.001

HLOS, hospital length of stay; ICULOS, intensive care unit length of stay; AKI, acute kidney injury; ARDS, acute respiratory distress syndrome; SSI, surgical site infection; VTE: venous thromboembolism; DVT, deep vein thrombosis; PE, pulmonary embolism; VAP, ventilator associated pneumonia; CAUTI: catheter associated urinary tract infection; CLABI: central line associated bloodstream infection; OR, operating room; PRBC, packed red blood cell.

© 2026 Huang W et al. *JAMA Surgery*.

eTable 8: the baseline of OS, failure of SAE and failure of OBS group					
Variables	Total (n = 4502)	OS (n = 3390)	SAE (n = 176)	OBS (n = 936)	p
Age, Median (IQR)	41 (28, 57)	40 (27, 56)	48 (30, 58)	45 (30, 58)	< 0.001
≥55, n (%)	1307 (29.0)	944 (27.8)	59 (33.5)	304 (32.5)	0.009
Sex, n (%)					
Male	3295 (73.2)	2483 (73.2)	133 (75.6)	679 (72.5)	0.700
Female	1207 (26.8)	907 (26.8)	43 (24.4)	257 (27.5)	
Race, n (%)					
White	3467 (77.0)	2584 (76.2)	136 (77.3)	747 (79.8)	0.103
Black	481 (10.7)	368 (10.9)	18 (10.2)	95 (10.1)	
Other	424 (9.4)	328 (9.7)	16 (9.1)	80 (8.5)	
Unknow	130 (2.9)	110 (3.2)	6 (3.4)	14 (1.5)	
BMI, n (%)					
<30	2934 (65.2)	2224 (65.6)	114 (64.8)	596 (63.7)	0.432
≥30	1277 (28.4)	940 (27.7)	54 (30.7)	283 (30.2)	
Unknow	291 (6.5)	226 (6.7)	8 (4.5)	57 (6.1)	
Hospital type, n (%)					
Non-Profit	3992 (88.7)	3010 (88.8)	161 (91.5)	821 (87.7)	< 0.001
Profit	485 (10.8)	364 (10.7)	10 (5.7)	111 (11.9)	
Government	23 (0.5)	16 (0.5)	5 (2.8)	2 (0.2)	
Unknow	2 (0.0)	0 (0.0)	0 (0.0)	2 (0.2)	
Payment, n (%)					
Insurance	2161 (48.0)	1636 (48.3)	89 (50.6)	436 (46.6)	0.346
Medicaid	889 (19.7)	663 (19.6)	36 (20.5)	190 (20.3)	
Self-Pay	584 (13.0)	454 (13.4)	14 (8.0)	116 (12.4)	
Medicare	444 (9.9)	319 (9.4)	16 (9.1)	109 (11.6)	
Other	322 (7.2)	244 (7.2)	16 (9.1)	62 (6.6)	
Unknow	102 (2.3)	74 (2.2)	5 (2.8)	23 (2.5)	
Bed size, n (%)					
<200	253 (5.6)	198 (5.8)	6 (3.4)	49 (5.2)	0.009
201 to 400	1121 (24.9)	854 (25.2)	42 (23.9)	225 (24.0)	
401 to 600	1278 (28.4)	998 (29.4)	41 (23.3)	239 (25.5)	
More than 600	1850 (41.1)	1340 (39.5)	87 (49.4)	423 (45.2)	
Trauma Level, n (%)					
I	2355 (52.3)	1736 (51.2)	103 (58.5)	516 (55.1)	0.270
II	1091 (24.2)	839 (24.7)	36 (20.5)	216 (23.1)	
III	10 (0.2)	8 (0.2)	0 (0.0)	2 (0.2)	
Unknow	1046 (23.2)	807 (23.8)	37 (21.0)	202 (21.6)	
ED first vital signs					
SBP, Median (IQR)	113 (94, 134)	110 (90, 131)	117 (100, 134)	123 (109, 141)	< 0.001
<90, n (%)	846 (18.8)	751 (22.2)	29 (16.5)	66 (7.1)	< 0.001
HR, Median (IQR)	101 (84, 121)	105 (86, 125)	97 (81, 113)	92 (79, 109)	< 0.001
≥120, n (%)	1220 (27.1)	1056 (31.2)	35 (19.9)	129 (13.8)	< 0.001

© 2026 Huang W et al. *JAMA Surgery*.

RR, Median (IQR)	20 (18, 24)	20 (18, 25)	20 (17, 24)	20 (18, 23)	< 0.001
GCS, Median (IQR)	15 (10, 15)	14 (7, 15)	15 (14, 15)	15 (14, 15)	< 0.001
Shock Index, Median (Q1, Q3)	0.89 (0.70, 1.16)	0.95 (0.74, 1.23)	0.84 (0.67, 1.04)	0.75 (0.61, 0.93)	< 0.001
Temperature, n (%)					
<36℃	694 (15.4)	559 (16.5)	26 (14.8)	109 (11.6)	< 0.001
≥36℃	2992 (66.5)	2156 (63.6)	122 (69.3)	714 (76.3)	
Unknow	816 (18.1)	675 (19.9)	28 (15.9)	113 (12.1)	
Pulse oximetry, n (%)					
<90%	482 (10.7)	411 (12.1)	9 (5.1)	62 (6.6)	< 0.001
≥90%	3932 (87.3)	2902 (85.6)	165 (93.8)	865 (92.4)	
Unknow	88 (2.0)	77 (2.3)	2 (1.1)	9 (1.0)	
Respiratory assistance, n (%)					
Yes	838 (18.6)	745 (22.0)	16 (9.1)	77 (8.2)	< 0.001
No	3599 (79.9)	2600 (76.7)	154 (87.5)	845 (90.3)	
Unknow	65 (1.4)	45 (1.3)	6 (3.4)	14 (1.5)	
ISS, Median (IQR)	35 (27, 43)	38 (29, 43)	34 (27, 42)	29 (22, 34)	< 0.001
≥25, n (%)	3920 (87.1)	3134 (92.4)	154 (87.5)	632 (67.5)	< 0.001
Comorbidities, n (%)					
Alcohol Use Disorder	341 (7.6)	239 (7.1)	18 (10.2)	84 (9.0)	0.057
Bleeding Disorder	35 (0.8)	19 (0.6)	3 (1.7)	13 (1.4)	0.011
Chemotherapy for Cancer	5 (0.1)	3 (0.1)	0 (0.0)	2 (0.2)	0.437
Congestive Heart Failure	56 (1.2)	30 (0.9)	4 (2.3)	22 (2.4)	0.002
Smoking	1215 (27.0)	833 (24.6)	60 (34.1)	322 (34.4)	< 0.001
Chronic Renal Failure	21 (0.5)	13 (0.4)	1 (0.6)	7 (0.7)	0.208
Cerebrovascular Accident	39 (0.9)	25 (0.7)	2 (1.1)	12 (1.3)	0.172
Diabetes Mellitus	355 (7.9)	243 (7.2)	15 (8.5)	97 (10.4)	0.005
Hypertension	856 (19.0)	599 (17.7)	41 (23.3)	216 (23.1)	< 0.001
COPD	165 (3.7)	105 (3.1)	8 (4.5)	52 (5.6)	0.002
Steroid Use	23 (0.5)	15 (0.4)	3 (1.7)	5 (0.5)	0.084
Cirrhosis	81 (1.8)	61 (1.8)	5 (2.8)	15 (1.6)	0.430
Dementia	23 (0.5)	12 (0.4)	1 (0.6)	10 (1.1)	0.031
Anticoagulant Therapy	177 (3.9)	119 (3.5)	12 (6.8)	46 (4.9)	0.020
Angina Pectoris	2 (0.0)	0 (0.0)	0 (0.0)	2 (0.2)	0.058
Mental disorder	428 (9.5)	301 (8.9)	27 (15.3)	100 (10.7)	0.007
Myocardial Infarction	23 (0.5)	17 (0.5)	1 (0.6)	5 (0.5)	0.774
Peripheral Arterial Disease	16 (0.4)	13 (0.4)	0 (0.0)	3 (0.3)	1.000
Substance Abuse Disorder	603 (13.4)	448 (13.2)	27 (15.3)	128 (13.7)	0.693
Any comorbidities	2514 (55.8)	1801 (53.1)	111 (63.1)	602 (64.3)	< 0.001
Mechanism, n (%)					
MVT	3751 (83.3)	2864 (84.5)	144 (81.8)	743 (79.4)	0.004
Fall	411 (9.1)	281 (8.3)	18 (10.2)	112 (12.0)	
Other	340 (7.6)	245 (7.2)	14 (8.0)	81 (8.7)	
Abdominal solid organ AIS, n (%)					

© 2026 Huang W et al. *JAMA Surgery.*

Liver AIS=2	845 (18.8)	698 (20.6)	17 (9.7)	130 (13.9)	< 0.001
Spleen					
AIS=3	1306 (29.0)	759 (22.4)	37 (21.0)	510 (54.5)	< 0.001
AIS=4	1566 (34.8)	1183 (34.9)	70 (39.8)	313 (33.4)	
AIS=5	1630 (36.2)	1448 (42.7)	69 (39.2)	113 (12.1)	
Kidney AIS=2	464 (10.3)	376 (11.1)	18 (10.2)	70 (7.5)	0.006
Pancreas AIS=2	255 (5.7)	230 (6.8)	5 (2.8)	20 (2.1)	< 0.001
AIS of each region, Median (IQR)					
Head	1 (0, 3)	1 (0, 3)	0 (0, 2)	0 (0, 2)	< 0.001
Face	0 (0, 1)	0 (0, 1)	0 (0, 1)	0 (0, 1)	0.015
Neck	0 (0, 0)	0 (0, 0)	0 (0, 0)	0 (0, 0)	0.225
Chest	3 (3, 3)	3 (3, 3)	3 (3, 3)	3 (3, 3)	< 0.001
Spine	0 (0, 2)	0 (0, 2)	0 (0, 2)	0 (0, 2)	0.010
Upper extremity	1 (0, 2)	1 (0, 2)	1 (0, 2)	1 (0, 2)	0.015
Lower extremity	1 (0, 3)	2 (0, 3)	2 (0, 3)	1 (0, 3)	0.003

BMI, Body Mass Index; SBP, systolic blood pressure; HR, heart rate; RR, respiratory rate; GCS, Glasgow Coma Scale; ISS, injury severity score; AIS, abbreviated injury score; ARDS, acute respiratory distress syndrome;

© 2026 Huang W et al. *JAMA Surgery*.

eTable 9: outcomes of OS, failure of SAE and failure of OBS group					
Variables	Total (n = 4502)	OS (n = 3390)	SAE (n = 176)	OBS (n = 936)	p
Mortality, n (%)	401 (8.9)	344 (10.1)	15 (8.5)	42 (4.5)	< 0.001
Hospital course, Median (IQR), d					
HLOS	12 (8, 21)	13 (8, 22)	15 (10, 23)	11 (8, 19)	< 0.001
ICULOS	6 (3, 13)	7 (3, 14)	7 (5, 14)	5 (3, 10)	< 0.001
Ventilator time	4 (2, 10)	4 (2, 10)	5 (3, 11)	4 (2, 11)	0.294
Complications, n (%)					
AKI	161 (3.6)	120 (3.5)	16 (9.1)	25 (2.7)	< 0.001
ARDS	130 (2.9)	109 (3.2)	5 (2.8)	16 (1.7)	0.051
Cardiac Arrest	170 (3.8)	135 (4.0)	7 (4.0)	28 (3.0)	0.367
Deep SSI	21 (0.5)	14 (0.4)	4 (2.3)	3 (0.3)	0.018
Organ Space SSI	34 (0.8)	25 (0.7)	5 (2.8)	4 (0.4)	0.013
Superficial SSI	22 (0.5)	15 (0.4)	2 (1.1)	5 (0.5)	0.263
Severe Sepsis	97 (2.2)	70 (2.1)	7 (4.0)	20 (2.1)	0.218
VTE	311 (6.9)	230 (6.8)	21 (11.9)	60 (6.4)	0.025
DVT	219 (4.9)	167 (4.9)	13 (7.4)	39 (4.2)	0.180
PE	123 (2.7)	84 (2.5)	9 (5.1)	30 (3.2)	0.071
Myocardial Infarction	27 (0.6)	22 (0.6)	1 (0.6)	4 (0.4)	0.737
Stroke	75 (1.7)	61 (1.8)	5 (2.8)	9 (1.0)	0.075
Unplanned Intubation	268 (6.0)	189 (5.6)	17 (9.7)	62 (6.6)	0.051
Unplanned Admission to ICU	291 (6.5)	146 (4.3)	27 (15.3)	118 (12.6)	< 0.001
Unplanned Visit to OR	332 (7.4)	192 (5.7)	46 (26.1)	94 (10.0)	< 0.001
CAUTI	41 (0.9)	29 (0.9)	5 (2.8)	7 (0.7)	0.049
CLABI	20 (0.4)	16 (0.5)	0 (0.0)	4 (0.4)	1.000
VAP	242 (5.4)	193 (5.7)	8 (4.5)	41 (4.4)	0.255
Alcohol Withdrawal Syndrome	51 (1.1)	34 (1.0)	5 (2.8)	12 (1.3)	0.056
Pressure Ulcer	125 (2.8)	91 (2.7)	7 (4.0)	27 (2.9)	0.527
Any Complications	1361 (30.2)	989 (29.2)	86 (48.9)	286 (30.6)	< 0.001
Transfusion, Mean (SD), ml					
PRBC	900 (1184)	1110 (1233)	534 (963)	211 (636)	< 0.001
Plasma	633 (928)	743 (963)	345 (721)	162 (559)	< 0.001
Platelet	106 (181)	123 (189)	70 (172)	29 (108)	< 0.001

HLOS, hospital length of stay; ICULOS, intensive care unit length of stay; AKI, acute kidney injury; ARDS, acute respiratory distress syndrome; SSI, surgical site infection; VTE: venous thromboembolism; DVT, deep vein thrombosis; PE, pulmonary embolism; VAP, ventilator associated pneumonia; CAUTI: catheter associated urinary tract infection; CLABI: central line associated bloodstream infection; OR, operating room; PRBC, packed red blood cell.

© 2026 Huang W et al. *JAMA Surgery.*

eTable 10: Effect size of SAE and OBS vs OS with mortality and any complication of other subgroups							
Groups	Mortality				Any complication		
	No.	HR (95%CI)	p		No.	OR (95%CI)	p
Shock index <1	403/9538				1580/9538		
OS	174/1880	Ref.			467/1880	Ref.	
SAE	60/1949	0.64 (0.47 to 0.87)	0.005		306/1949	0.84 (0.70, 1.00)	0.056
OBS	169/5709	0.58 (0.45 to 0.75)	<0.001		807/5709	0.81 (0.69, 0.96)	0.012
Shock index ≥1	283/3392				969/3392		
OS	170/1510	Ref.			522/1510	Ref.	
SAE	28/588	0.56 (0.37 to 0.86)	0.008		135/588	0.64 (0.51, 0.82)	<0.001
OBS	85/1294	0.63 (0.46 to 0.85)	0.003		312/1294	0.73 (0.59, 0.89)	0.002
Spleen AIS=3	334/6733				1192/6733		
OS	114/759	Ref.			262/759	Ref.	
SAE	35/889	0.52 (0.35 to 0.78)	0.001		159/889	0.64 (0.50, 0.83)	<0.001
OBS	185/5085	0.54 (0.41 to 0.70)	<0.001		771/5085	0.60 (0.49, 0.73)	<0.001
Spleen AIS=4	185/3775				741/3775		
OS	106/1183	Ref.			316/1183	Ref.	
SAE	34/1148	0.56 (0.37 to 0.84)	0.006		187/1148	0.67 (0.54, 0.84)	<0.001
OBS	45/1444	0.64 (0.44 to 0.93)	0.021		238/1444	0.75 (0.61, 0.93)	0.009
Spleen AIS=5	167/2422				616/2422		
OS	124/1448	Ref.			411/1448	Ref.	
SAE	19/500	1.00 (0.60 to 1.69)	0.986		95/500	0.87 (0.66, 1.15)	0.325
OBS	24/474	0.99 (0.61 to 1.60)	0.969		110/474	1.16 (0.89, 1.52)	0.274

HR, hazard ratio; OR, odds ratio.

© 2026 Huang W et al. *JAMA Surgery*.

eTable 11: Effect size of SAE and OBS vs OS with transfusion using multivariable linear regression analysis.				
Outcome	No.	Mean (SD)	Multivariate linear regression	
			β(95%CI)	p
PRBC, ml	12861	449(889)		
OS	3364	1110(1233)	Ref.	
SAE	2522	310(608)	-569 (-610, -529)	<0.001
OBS	6975	180(546)	-639 (-676, -603)	<0.001
Plasma, ml	9266	339(714)		
OS	2988	743(963)	Ref.	
SAE	1856	178(437)	-406 (-444, -368)	<0.001
OBS	4422	135(445)	-415 (-449, -381)	<0.001
Platelet, ml	9207	55(139)		
OS	2936	124(190)	Ref.	
SAE	1854	30(101)	-68 (-75, -60)	<0.001
OBS	4417	19(85)	-72 (-79, -65)	<0.001

PRBC, packed red blood cell.

© 2026 Huang W et al. *JAMA Surgery*.

eTable 12：Univariable and multivariable logistic regression analysis for risk factors of failure				
Variables	Univariable logistic regression		Multivariable logistic regression	
	OR (95% CI)	p	OR (95% CI)	p
Age	1.01 (1.01 to 1.01)	< 0.001	1.01 (1.01 to 1.01)	< 0.001
Sex				
Male	Ref.			
Female	0.92 (0.80 to 1.05)	0.22		
Race				
Black	Ref.			
White	1.08 (0.88 to 1.33)	0.44		
Other	0.86 (0.65 to 1.14)	0.29		
BMI				
<30	Ref.			
≥30	0.99 (0.86 to 1.13)	0.83		
Payment				
Medicaid	Ref.			
Self-Pay	0.86 (0.67 to 1.07)	0.18		
Insurance	0.87 (0.74 to 1.03)	0.10		
Medicare	1.13 (0.90 to 1.42)	0.29		
Bed size				
<200	Ref.			
201 to 400	1.01 (0.75 to 1.37)	0.93		
401 to 600	0.89 (0.66 to 1.19)	0.43		
More than 600	1.24 (0.93 to 1.64)	0.15		
Trauma Level				
I	Ref.		Ref.	
II	0.79 (0.68 to 0.92)	0.002	0.78 (0.66 to 0.91)	0.002
SBP				
<90 mmHg	Ref.		Ref.	
≥90 mmHg	1.37 (1.11 to 1.70)	0.003	1.35 (1.08 to 1.68)	0.007
HR				
<120	Ref.		Ref.	
≥120	0.68 (0.58 to 0.81)	< 0.001	0.81 (0.68 to 0.97)	0.023
RR	0.99 (0.98 to 1.00)	0.08		
GCS	1.05 (1.04 to 1.07)	< 0.001	1.04 (1.02 to 1.05)	< 0.001
Pulse Oximetry				
<90%	Ref.			
≥90%	1.28 (1.00 to 1.62)	0.05		
Respiratory assistance				
No	Ref.			
Yes	0.60 (0.48 to 0.74)	< 0.001		
Alcohol Use Disorder				
No	Ref.		Ref.	

© 2026 Huang W et al. *JAMA Surgery*.

Yes	1.54 (1.25 to 1.91)	< 0.001	1.41 (1.13 to 1.75)	0.002
Chemotherapy for Cancer				
No	Ref.			
Yes	1.30 (0.30 to 5.66)	0.726		
Congestive Heart Failure				
No	Ref.		Ref.	
Yes	2.38 (1.58 to 3.6)	< 0.001	1.68 (1.09 to 2.60)	0.019
Smoking				
No	Ref.		Ref.	
Yes	1.46 (1.29 to 1.67)	< 0.001	1.41 (1.23 to 1.61)	< 0.001
Chronic Renal Failure				
No	Ref.			
Yes	1.30 (0.62 to 2.72)	0.48		
Diabetes Mellitus				
No	Ref.			
Yes	1.29 (1.05 to 1.59)	0.02		
Hypertension				
No	Ref.			
Yes	1.33 (1.15 to 1.54)	< 0.001		
Steroid Use				
No	Ref.			
Yes	1.39 (0.66 to 2.91)	0.38		
Cirrhosis				
No	Ref.			
Yes	1.38 (0.88 to 2.16)	0.16		
Anticoagulant Therapy				
No	Ref.		Ref.	
Yes	1.84 (1.39 to 2.43)	< 0.001	1.33 (0.98 to 1.80)	0.06
Mechanism				
MVT	Ref.			
Fall	1.17 (0.92 to 1.35)	0.26		
Other	1.05 (0.84 to 1.31)	0.67		
Abdominal solid organ AIS, n (%)				
Liver				
0	Ref.			
2	0.99 (0.83 to 1.19)	0.95		
Spleen				
3	Ref.		Ref.	
4	1.28 (1.19 to 1.47)	< 0.001	1.31 (1.14 to 1.51)	< 0.001
5	0.99 (0.83 to 1.17)	0.89	1.07 (0.90 to 1.27)	0.50
Kidney				
0	Ref.			
2	0.84 (0.67 to 1.05)	0.13		

© 2026 Huang W et al. *JAMA Surgery*.

Pancreas				
0	Ref.			
2	0.96 (0.65 to 1.42)	0.84		
AIS of each region, Median (IQR)				
Head	0.92 (0.88 to 0.96)	< 0.001		
Face	0.93 (0.85 to 1.01)	0.07		
Neck	1.07 (0.97 to 1.19)	0.16		
Chest	0.97 (0.92 to 1.03)	0.31		
Spine	0.98 (0.93 to 1.04)	0.54		
Upper Extremity	1.00 (0.94 to 1.07)	0.98		
Lower Extremity	1.01 (0.96 to 1.05)	0.78		

BMI, Body Mass Index; SBP, systolic blood pressure; HR, heart rate; RR, respiratory rate; GCS, Glasgow Coma Scale; AIS, abbreviated injury score.

© 2026 Huang W et al. *JAMA Surgery*.

eFigure: Patient selection flow diagram

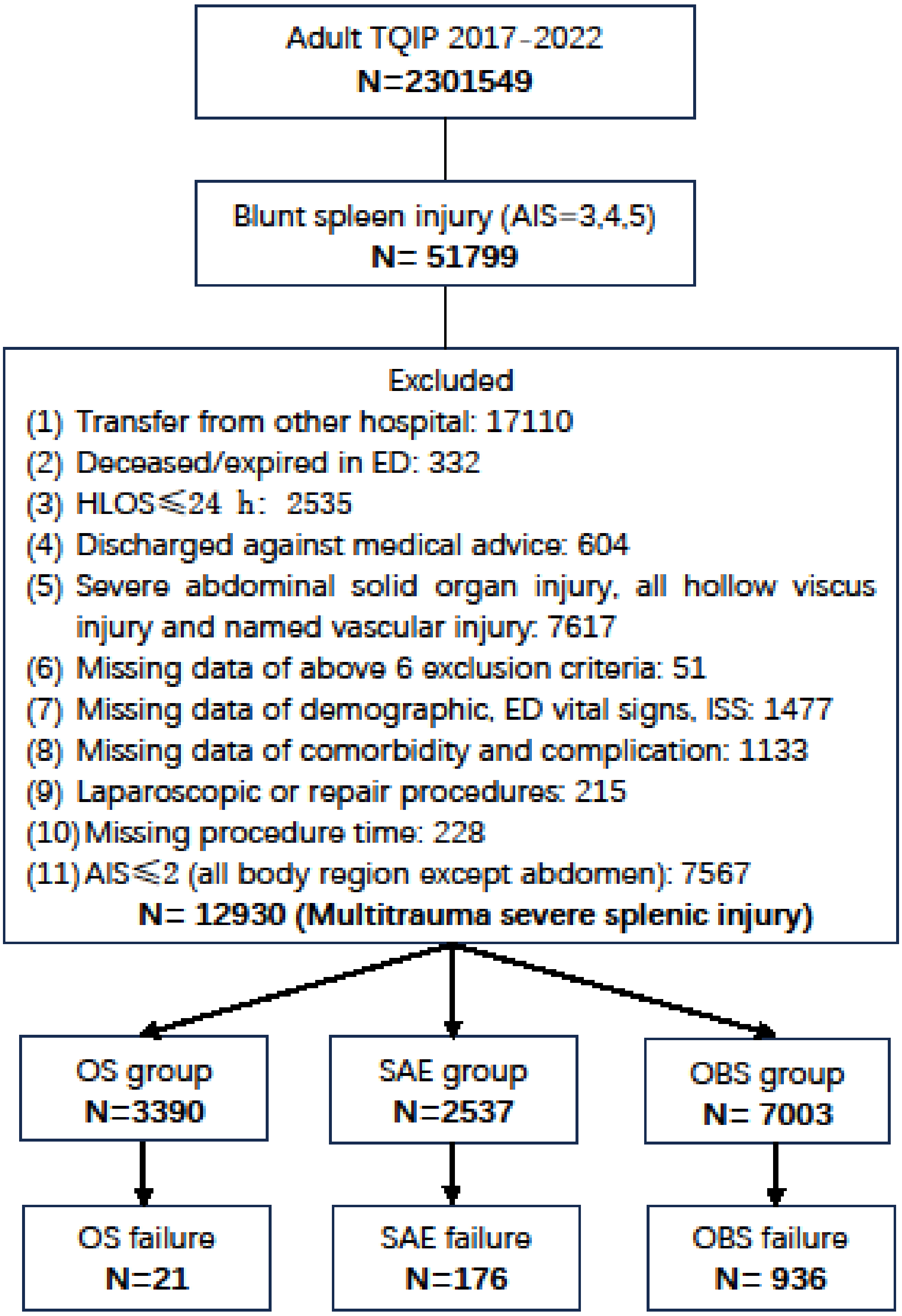

TQIP, Trauma Quality Improvement Program; AIS, abbreviated injury scale; ED, emergency department; HLOS, Hospital Length of Stay; ISS, Injury Severity Score

© 2026 Huang W et al. *JAMA Surgery.*
