# Supplementary material for: Severe Splenic Injuries in Patients With Multiple Trauma
Source: JAMA Surg. 2026 Feb 25;161(4):413–23. doi: 10.1001/jamasurg.2026.0016 (PMC12936967; doi:10.1001/jamasurg.2026.0016)
Supplement: Supplement 2. — Data sharing statement [file jamasurg-e260016-s002.pdf]

Data Sharing Statement

Huang. Severe Splenic Injuries in Patients With Multiple Trauma. *JAMA Surg*. Published February 25, 2026. doi:10.1001/jamasurg.2026.0016

Data
Data available: No
